# Prevalence and genotype distribution of norovirus and rotavirus among children under five with diarrheal infection in lower-middle-income countries after *Rotavirus* vaccine introduction: A systematic review and meta-analysis

**DOI:** 10.1371/journal.pone.0359886

**Published:** 2026-10-13

**Authors:** Firayad Ayele, Tesfaye Assebe, Miheret Mandefro, Desalegn Admassu Ayana, Nega Assefa

**Affiliations:** College of Health and Medical Sciences, Haramaya University, Harar, Ethiopia; Federal University of Sergipe, BRAZIL

## Abstract

**Background:**

Rotavirus and norovirus are the leading viral causes of acute diarrheal disease worldwide. Both viruses encompass multiple genotypes and subgenotypes, and this genetic diversity presents a significant challenge to rotavirus vaccine effectiveness and development. High-quality evidence on the prevalence and circulating genotypes of these enteric viruses among children under five years of age remains limited, particularly following the introduction of rotavirus vaccination in low and middle-income countries (LMICs). Therefore, this systematic review and meta-analysis aimed to synthesizes available evidence on the prevalence and genotype distribution of norovirus and rotavirus in this population.

**Methods:**

A systematic literature search was conducted across five major databases PubMed, Scopus, ScienceDirect, Embase, and Web of Science between February 20 and 28, 2026. Duplicate removal, title and abstract screening, full-text review and data extraction were performed independently by two investigators using the Rayyan web-based platform. STATA version 17 software was used for statistical analysis. A random-effects model was applied to estimate pooled prevalence and effect sizes. Heterogeneity was evaluated using Cochran’s Q statistic, the I^2^ statistic, forest plots, and Galbraith plots. Publication bias was assessed using both graphical (funnel plots) and statistical methods.

**Results:**

A total of 35,704 children under five years with diarrheal infection from LMICs were included across eligible studies. The overall pooled prevalence of norovirus and rotavirus combined was 18% (95% CI: 15–21%). Rotavirus had a higher pooled prevalence than norovirus, at 24% (95% CI: 19.6–28.3%) and 13% (95% CI: 11–15%), respectively. Substantial heterogeneity was observed across studies (I^2^ = 98.72%, p < 0.001). Among norovirus genotypes, GII was predominant (76%), with subgenotype GII.4 accounting for 53% of cases, followed by GI (23%). The most frequently identified rotavirus genotypes were G3[P8] (39%) and G1[P8] (17%).

**Conclusions:**

Norovirus and rotavirus remain significant causes of diarrheal disease among children under five in LMICs, even after the introduction of rotavirus vaccination. Approximately one in five children with diarrheal illness were infected with at least one of these virusesGII, particularly subgenotype GII.4, was the dominant norovirus strain, while G3[P8] and G1[P8] were the most prevalent rotavirus genotypes. These findings underscore the need to strengthen surveillance systems, consider circulating genotypes in vaccine formulation, and continuously monitor genotype shifts within the population.

## Introduction

Viral diarrheal infection, also referred to as viral gastroenteritis, is a condition characterized by the frequent passage of watery, loose stools caused by a pathogenic virus [1]. The primary viral agents responsible for this condition are rotavirus, which predominantly affects children, and norovirus, which can infect individuals of all age groups [2,3].

Rotavirus is characterized as a non-enveloped, double-stranded RNA virus containing 11 gene segments [4]. These segments encode six structural proteins, designated VP1-VP4 and VP6-VP7 [5,6]. Additionally, the rotavirus genome encodes six non-structural proteins (NSP1 to NSP6) [7] and there are nine antigenically distinct groups (A through J) [8]. Geno-typically, rotavirus is classified into 36 G types and 51 P types based on the VP7 glycoprotein and VP4 protease-sensitive protein. The most prevalent strains causing severe diarrhea in humans are G1P, G2P, G3P, G4P, and G9P, with G1-G4, G9, and G12 being the most common G genotypes and P[4], P[6], and P[8] the most frequently observed P genotypes worldwide [9]. However, with the introduction of four rotaviruses A vaccine, the incidence of disease caused by rotavirus has decreased significantly, although norovirus remains a leading cause of gastroenteritis [4,10,11].

Norovirus (NoV) belongs to the Caliciviridae family and is a single stranded, non- enveloped, positive sense virus [12,13]. The genome of this virus categorized into six Geno groups, with Geno groups I and II (GI and GII) accounting for most human infections [14]. Each Geno group can be subdivided into various genotypes, with GI consisting of at least 9 genotypes and GII containing around 27(GII.1–GII.27) [15]. The majority of human NoV infections are attributed to GII strains, particularly the GII.4 genotype, which is responsible for approximately 55–85% of clinical cases globally [16].

Diarrheal disease is the second most prevalent illness and the third leading cause of death among children under five, resulting in approximately 443,832 deaths and 1.7 billion cases annually [17]. In lower- and middle-income countries, diarrhea is responsible for about 25% to 30% of deaths in children, leading to high mortality rates, morbidity, and economic crises. Notably, over 70% of diarrhea-related fatalities in children under five occur in Africa [18], with the majority of morbidity and mortality concentrated in middle income countries, where 88% of these cases are related to unsafe water, inadequate sanitation, and poor hygiene [19,20].

Viral gastroenteritis has a substantial effect on patients, especially on children under five. Severe diarrheal episodes can lead to dehydration and loss of water and electrolytes (sodium, chloride, potassium, bicarbonate), which may be fatal in young children. Additionally, children under five often experience concurrent malnutrition and weight loss [18]. These health consequences impose economic burdens on families, healthcare systems, and negatively affect national productivity, alongside increasing morbidity and mortality. In low- and middle-income countries, factors such as low maternal education, poor sanitation, contaminated water, short breastfeeding duration, inadequate handwashing, lack of rotavirus vaccination, improper feces disposal, young child age, and poor food hygiene further worsen the problem [17,19,21].

In LMICs, to the best of our knowledge there is no high-quality evidence on the pooled prevalence and genotypes distribution of rotavirus and norovirus among under five children, especially following the introduction of rotavirus vaccines. Filling this gap would improve understanding of the molecular epidemiology and pooled prevalence information that is crucial for outbreak preparedness and response and for guiding the development of vaccines targeting the most common genotypes. Therefore, the present study, aimed to systematically review and quantitatively synthesize evidence on prevalence and genotype distribution of rotavirus and norovirus among children under five with diarrheal infection in lower-middle-income countries following the introduction of rotavirus vaccination.

The findings of this study provide valuable evidence for low and middle-income countries, health professionals, NGOs, policymakers, and other stakeholders involved in childhood diarrheal disease. The findings also serve as a baseline for future research and vaccine development.

## Methods and materials

### Registration and reporting

This systematic review and meta-analysis was carried out according to the protocol registered in the International Prospective Register of Systematic Reviews (PROSPERO), registration number CRD420261 349950 (https://www.crd.york.ac.uk/PROSPERO/viewrecord.php?ID=CRD420261349950).

### Data source and study search strategy

A comprehensive search for relevant literature was conducted across various online databases, including PubMed, Scopus, Science Direct, Embase, and Web of Science. The search was done from February 20–28/2026 using the searching terms included “Rotavirus, Norovirus, Calicivirus, “Norwalk virus”, “viral diarrhea”, “enteric viruses” Genotype, prevalence, “Under-Five Children”, “pediatric gastroenteritis”, diarrhea” without any filters applied for date. Boolean operators such as AND, OR were utilized to refine the search results (the detail S1 Table).

### Inclusion and exclusion criteria for studies

Studies were eligible for inclusion if they met all of the following criteria: (1) conducted in low- and middle-income countries (LMICs; see S2 Table for the full list of eligible countries); (2) for rotavirus-focused articles, published after the national introduction of the rotavirus vaccine in the respective country; (3) enrolled children under five years of age as the study population; (4) used stool specimens for laboratory diagnosis; (5) included a minimum sample size of 50 participants; (6) published in the English language; (7) employed a case-control, cross-sectional or large scale surveillance design; (8) reported the prevalence or genotype distribution of rotavirus, norovirus, or both; (9) studies with full-text articles; and (10) achieved a quality assessment score greater than 50% (moderate quality or above) on the JBI critical appraisal checklist.

Studies were excluded if they were case reports, clinical trials, editorials, commentaries, meta-analyses, previously published systematic reviews, or conference abstracts lacking full-text availability.

### Study selection

Upon completing the literature search across various databases, the search results were imported into Rayyan (AI-Powered Systematic Review Management Platform, https://www.rayyan.ai/). Following the Preferred Reporting Items for Systematic Reviews and Meta-Analyses (PRISMA)(S3 Table) format and studies screening, duplicate removal, title/abstract screening, and full-text screening was conducted by two independent authors. Any conflict during screening discussed in detail to reach a consensus on whether to accept or reject the articles for inclusion in the report.

### Data extraction

Eligible articles were extracted using a Microsoft Excel spread-sheet by two authors. The full texts were thoroughly reviewed to ensure that all relevant information required for the analysis was captured. Each study was assigned a unique identification code to prevent duplication and misclassification. After the full text review, the authors extracted information such as the first Authors name, year of publication, country, study design, detection methods, sample size, event/cases, prevalence, outcome (rotavirus or norovirus), and genogroup/genotype.

### Study risk of bias and quality assessment

The overall quality of each eligible article was evaluated by two independent authors. A standardized checklist developed by the Joanna Briggs Institute (JBI) [22] specifically for assessing the quality of prevalence studies was used. This checklist included nine main criteria, each criterion could be answered Yes (Y), No (N), unclear (U) and not applicable (NA). An overall quality assessment score was assigned to each study based on the answers to these nine questions. The overall quality of the studies was categorized as ‘high’ (scores ≥8 points), ‘moderate’ (scores >5 to <8 points), or ‘low’ (scores ≤5 points).

### Evidence synthesis and analysis

All the extracted data are qualitatively described and presented in textual description and also presented in tables and Figs. For quantitative analysis STATA version 17 software was used, and the pooled prevalence of norovirus, rotavirus and their genotype was estimated using a Restricted Maximum Likelihood (REML) random-effects model with corresponding 95% confidence intervals. Studies reporting zero prevalence were retained in the meta-analysis and were not excluded on the basis of a zero event. Heterogeneity across the included studies was assessed using a Q statistic, I² statistic, tau-squared (τ²), forest plot & Galbraith plot, where I² statistic values of 0%, < 25%, 25–75%, and >75% indicated no, low, moderate, and high heterogeneity, respectively. Publication bias was evaluated using funnel plots, egger’s test, begg test & contour-enhanced funnel plot. A leave-one-out sensitivity analysis was also performed to assess the robustness of the pooled estimates.

## Results

### Study selection process

A total of 13,031 articles were identified from various electronic databases. Of these, 3,606 were removed as duplicates, leaving 9425 articles for title and abstract screening. After screening, 265 articles were selected for further assessment; full text could not be retrieved for 5 articles. Therefore, 260 articles go through full-text screening, and 215 were excluded for not meeting the predetermined eligibility criteria. Finally, 45 articles were included in the data extraction process (Fig 1).

**Fig 1:**
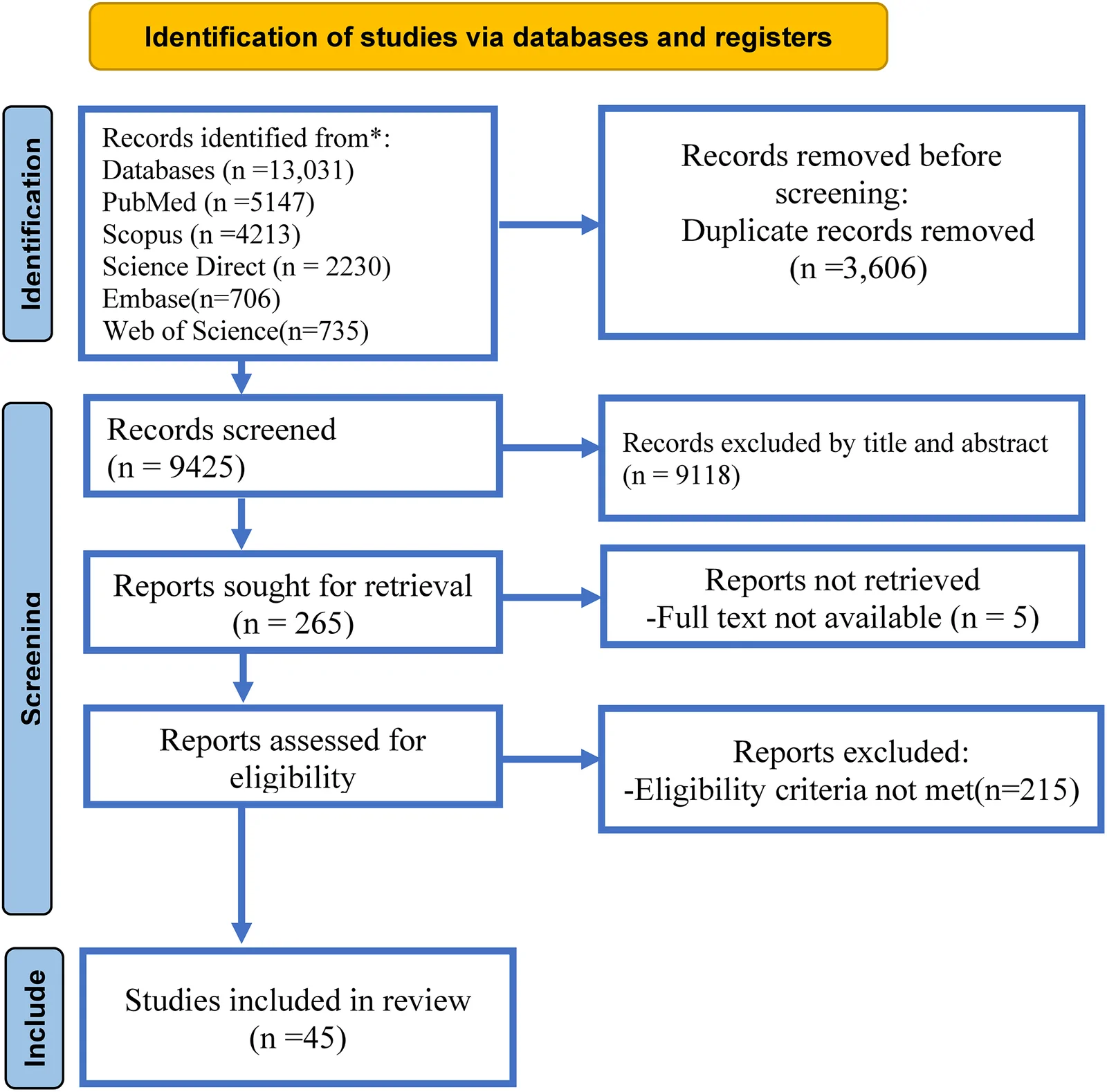
PRISMA flow diagram showing the selection process of studies for systematic review and meta-analysis on prevalence and genotype distribution of norovirus and rotavirus among children under five with diarrheal infection in lower-middle-income countries after rotavirus vaccine introduction, 2026.

### Risk of bias and quality assessment

The risk of bias and methodological quality of the included studies were assessed using the standardized Joanna Briggs Institute (JBI) Critical Appraisal Checklist for Studies Reporting Prevalence Data [22].. All 45 articles were assessed; quality scores ranged from 55.5% (moderate) to 100% (high), with the majority of studies attaining high quality scores. Based on this assessment, all articles were considered valid and were included in the review (S4 Table).

### Characteristics of included studies

A total of 45 eligible studies were included in evidence synthesis. All studies are reported from lower and middle-income countries. Among 45 studies, 43 studies were considered for genotype distribution. Across these studies, the total number of study participant (children under five years of age) found to be 35,704. For articles reporting norovirus, no publication-year restrictions were applied. For studies reporting rotavirus, we restricted inclusion to those conducted from the rotavirus vaccine introduction year onward; accordingly, studies from 2006–2025 were included. Across the included studies, norovirus prevalence ranged from 3.12% to 30.50%, while rotavirus prevalence ranged from 17.90% to 46.50%%.

India contributed the largest number of included studies, with a total of 20 studies [23–41] followed by 4 studies each from Bangladesh [42–45] and Ethiopia [46–49]; 3 studies from Nigeria [50–52]; two studies from Honduras [53] and Morocco [54]; and one study each from Angola [55], Bhutan [56], Cameroon [57], Gabon [58], Kenya [59], Lebanon [60], Nepal [61], Pakistan [62], Senegal [63], and Zambia [64].

Of the 45 studies included, most (34) were cross-sectional while 11 were large-scale surveillance studies. In terms of detection methods, 23 studies used RT‑PCR 19 studies used both RT‑PCR and ELISA 3 studies used ELISA and multiplex RT‑PCR. Sample sizes for detection ranged from 55 to 5,408. Regarding outcome variable, 24 studies reported *norovirus* as an outcome variable, 21 reporting rotavirus outcomes and two studies reported both viruses (Table 1).

**Table 1:** Summary characteristics of the 45 study reports for the systematic review and meta-analysis on prevalence and genotype distribution of norovirus and rotavirus among children under five with acute diarrhea in lower-middle-income countries after rotavirus vaccine introduction, 2026.

No	Authors name, year	Country	Study Design	Region	Detection Methods	Sample size	Event/Cases	Total prevalence	Outcome
1.	Abubakar et al,2023	Nigeria	Cross-sectional	West Africa	RT‐PCR	200	42	21%	Norovirus
2.	Alam et al, 2015	Pakistan	Cross-sectional	South Asia	RT‐PCR	255	41	16.10%	Norovirus
3.	Arowolo, K.O, et al, 2023	Nigeria	Cross-sectional	West Africa	RT‐PCR	175	13	7.40%	Norovirus
4.	S.K. Dey et al, 2007	Bangladesh	Cross-sectional	South Asia	RT‐PCR	917	41	4.50%	Norovirus
5.	El Qazoui et a, 2024	Morocco	Cross-sectional	North Africa	ELISA, multiplex PCR	335	89	26.60%	Rotavirus
6.	El Qazoui et a, 2024	Morocco	Cross-sectional	North Africa	ELISA, multiplex PCR	335	54	16.10%	Norovirus
7.	A. Esteves et al, 2018	Angola	Cross-sectional	Southern Africa	RT‐PCR	343	58	17.40%	Norovirus
8.	A. Gelaw et al, 2019	Ethiopia	Cross-sectional	East Africa	RT‐PCR	450	60	13.30%	Norovirus
9.	T.N. Hoa-Tran et al,2015	Nepal	Cross-sectional	South Asia	RT‐PCR	4437	356	8%	Norovirus
10.	Gopalkrishna et al,2021	India	Cross-sectional	South Asia	ELISA, RT‐PCR	308	44	14.30%	Norovirus
11.	Gopalkrishna et al,2021	India	Cross-sectional	South Asia	ELISA, RT‐PCR	308	94	30.50%	Rotavirus
12.	Japhet et al, 2012	Nigeria	Cross-sectional	West Africa	RT‐PCR	55	14	25.50%	Norovirus
13.	Kebe et al, 2021	Senegal	Surveillance	West Africa	RT‐PCR	599	79	13.20%	Norovirus
14.	R. Kulkarni et al, 2016	India	Cross-sectional	South Asia	RT‐PCR	1712	126	7.40%	Norovirus
15.	S.E. Lekana-Douki et al, 2015	Gabon	Cross-sectional	Central Africa	RT‐PCR	317	73	23.40%	Norovirus
16.	M. Lo et al,2021	India	Cross-sectional	South Asia	RT‐PCR	2812	170	6.04%	Norovirus
17.	Melhem NM et al., 2016	Lebanon	Cross-sectional	Middle East	RT‐PCR	739	83	11.20%	Norovirus
18.	Menon et al, 2013	India	Cross-sectional	South Asia	RT‐PCR	282	28	9.90%	Norovirus
19.	Mugyia et al., 2019	Cameroon	Cross-sectional	Central Africa	RT‐PCR	902	76	8.40%	Norovirus
20.	Nataraju et al, 2010	India	Surveillance	South Asia	RT‐PCR	2495	78	3.12%	Norovirus
21.	Ortiz-Quintero et al,2024	Honduras	Cross sectional	Central America	RT‐PCR	100	13	13%	Norovirus
22.	Satter et al 2022	Bangladesh	surveillance	South Asia	RT‐PCR	1008	137	14%	Norovirus
23.	N. Sharif et al, 2023	Bangladesh	Cross sectional	South Asia	RT‐PCR	404	27	6.70%	Norovirus
24.	Wangchu et al, 2017	Bhutan	Cross sectional	South Asia	RT‐PCR	623	147	23.60%	Norovirus
25.	A. Abebe et a, 2018	Ethiopia	Surveillance	East Africa	ELISA, RT‐PCR	815	161	20.00%	Rotavirus
26.	Kumar et al, 2022	India	Cross sectional	South Asia	ELISA, RT‐PCR	240	35	14.58%	Rotavirus
27.	Namboodiripad1, 2025	India	Cross sectional	South Asia	ELISA, RT‐PCR	643	115	17.90%	Rotavirus
28.	Kumar, et al, 2025	India	Cross-sectional	South Asia	ELISA, RT‐PCR	510	237	46.50%	Rotavirus
29.	Nyamanga et al, 2024	Kenya	Cross sectional	East Africa	ELISA, multiplex PCR	200	23	11.50%	Rotavirus
30.	Singh et al, 2025	India	Cross-sectional	South Asia	ELISA, RT‐PCR	461	165	35.80%	Rotavirus
31.	Gurung, et al, 2025	India	Cross sectional	South Asia	ELISA, RT‐PCR	200	40	20.00%	Rotavirus
32.	jafet et al, 2024	Honduras	Cross sectional	Central America	ELISA, RT‐PCR	100	14	14.00%	Rotavirus
33.	jain, et al, 2021	India	Surveillance	South Asia	ELISA, RT‐PCR	734	95	12.94%	Rotavirus
34.	Simwaka et al., 2021	Zambia	Surveillance	Southern Africa	ELISA, RT‐PCR	424	153	36.00%	Rotavirus
35.	Engade et al, 2025	India	Surveillance	South Asia	ELISA, RT‐PCR	534	173	32.70%	Rotavirus
36.	Mohanty et al, 2025	India	Surveillance	South Asia	ELISA, RT‐PCR	5408	1870	34.60%	Rotavirus
37.	Mohanty et alb, 2025	India	Surveillance	South Asia	ELISA, RT‐PCR	288	67	23.30%	Rotavirus
38.	Rajani et al, 2025	India	Cross-sectional	South Asia	ELISA, RT‐PCR	405	97	23.90%	Rotavirus
39.	Rawal et al, 2021	India	Surveillance	South Asia	ELISA, RT‐PCR	444	107	24.10%	Rotavirus
40.	Pradhan, et al, 2021	India	Cross-sectional	South Asia	ELISA, RT‐PCR	1963	715	36.40%	Rotavirus
41.	Vyas et al, 2025	India	Surveillance	South Asia	ELISA, RT‐PCR	1156	206	17.90%	Rotavirus
42.	India et al 2025	India	Cross-sectional	South Asia	ELISA, RT‐PCR	114	13	11.40%	Rotavirus
43.	Redda et al. 2025	Ethiopia	Cross sectional	East Africa	RT‐PCR	247	30	12.14%	Rotavirus
44.	Rahman et at.,2016	Bangladesh	surveillance	South Asia	RT‐PCR	547	149	27%	Norovirus
45.	Tegegne D, et al,2024	Ethiopia	Cross-sectional	East Africa	RT‐PCR	160	20	12.5%	Norovirus

ELISA=Enzyme*-*Linked Immunosorbent Assay, RT‐PCR =Reverse Transcription Polymerase Chain Reaction

### Pooled prevalence of *Norovirus* and *Rotavirus*

The pooled prevalence of norovirus and rotavirus among children under five with diarrheal infection in LMICs was 18% (95% CI: 15–21%), with substantial heterogeneity (I² = 98.85%, p < 0.001) (Fig 2).

**Fig 2:**
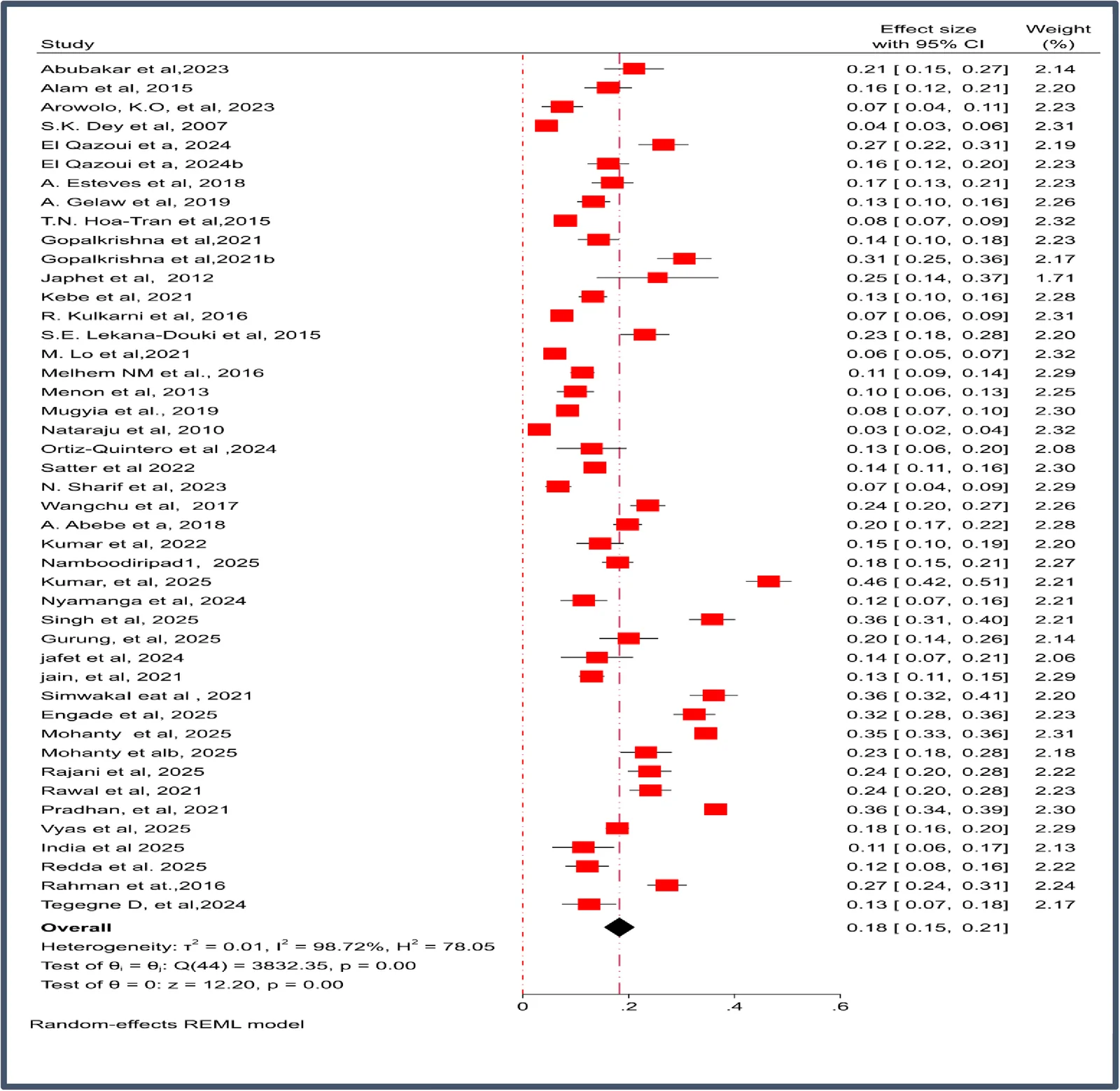
Forest plot showing the pooled prevalence of norovirus and rotavirus among under five children with diarrheal infection in LMICs.

### Subgroup analysis, sensitivity analysis, and publication bias

In this systematic review and meta-analysis, the results of Cochran’s Q test (3832.35, df = 44, p < 0.001), tau-squared (τ² = 0.0096), and particularly the I² statistic (98.72%) revealed substantial and statistically significant heterogeneity, which was further illustrated by the Galbraith plot (Fig 3) and the forest plot (Fig 4). Accordingly, subgroup analyses were performed based on outcome (norovirus vs rotavirus), region, study design, and detection methods. In addition, a meta-regression analysis was conducted to examine the effect of sample size.

**Fig 3:**
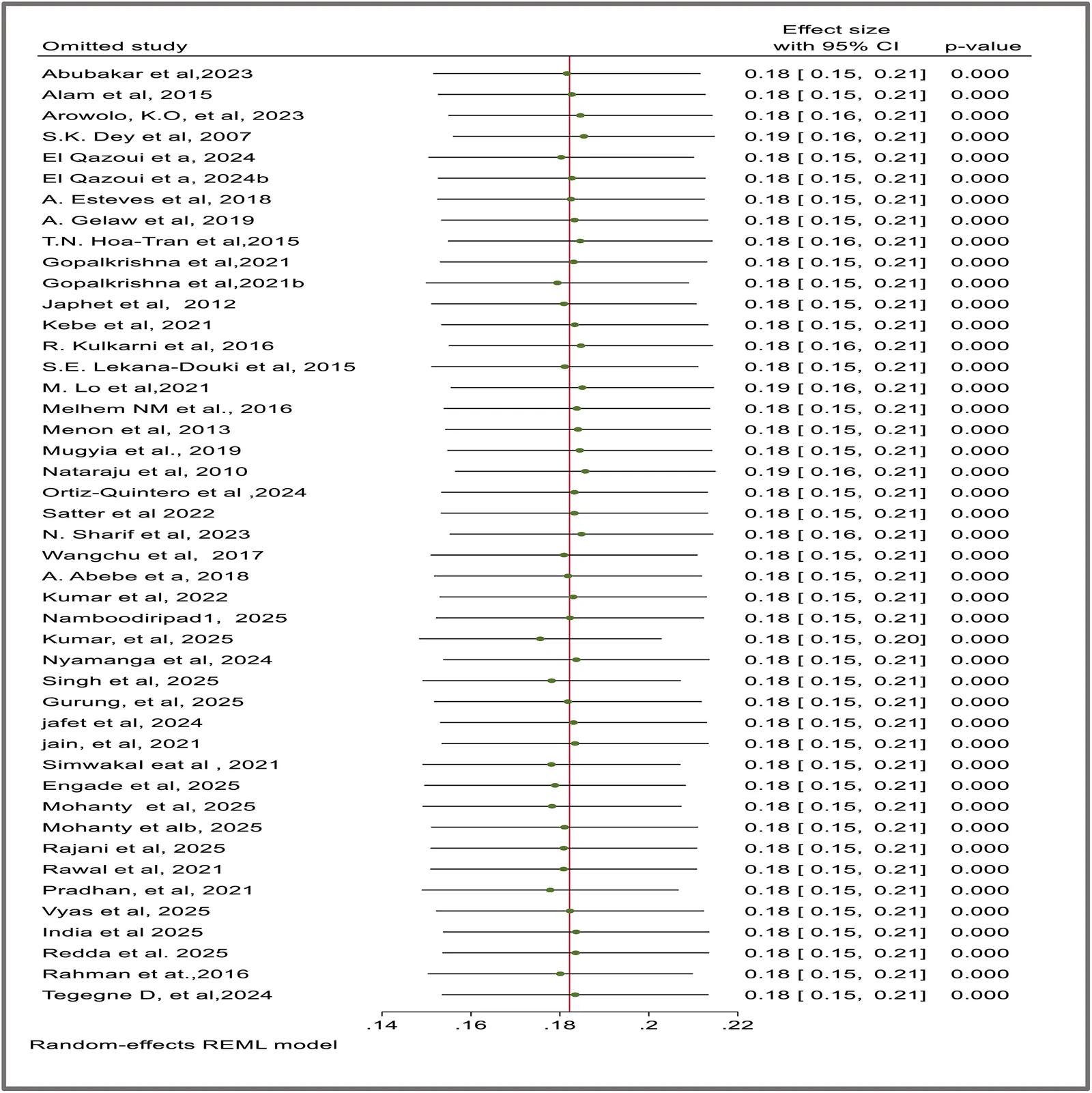
Forest plot showing leave-one-out meta-analysis for the pooled prevalence of norovirus and rotavirus among under five children with diarrheal infection in LMICs.

**Fig 4:**
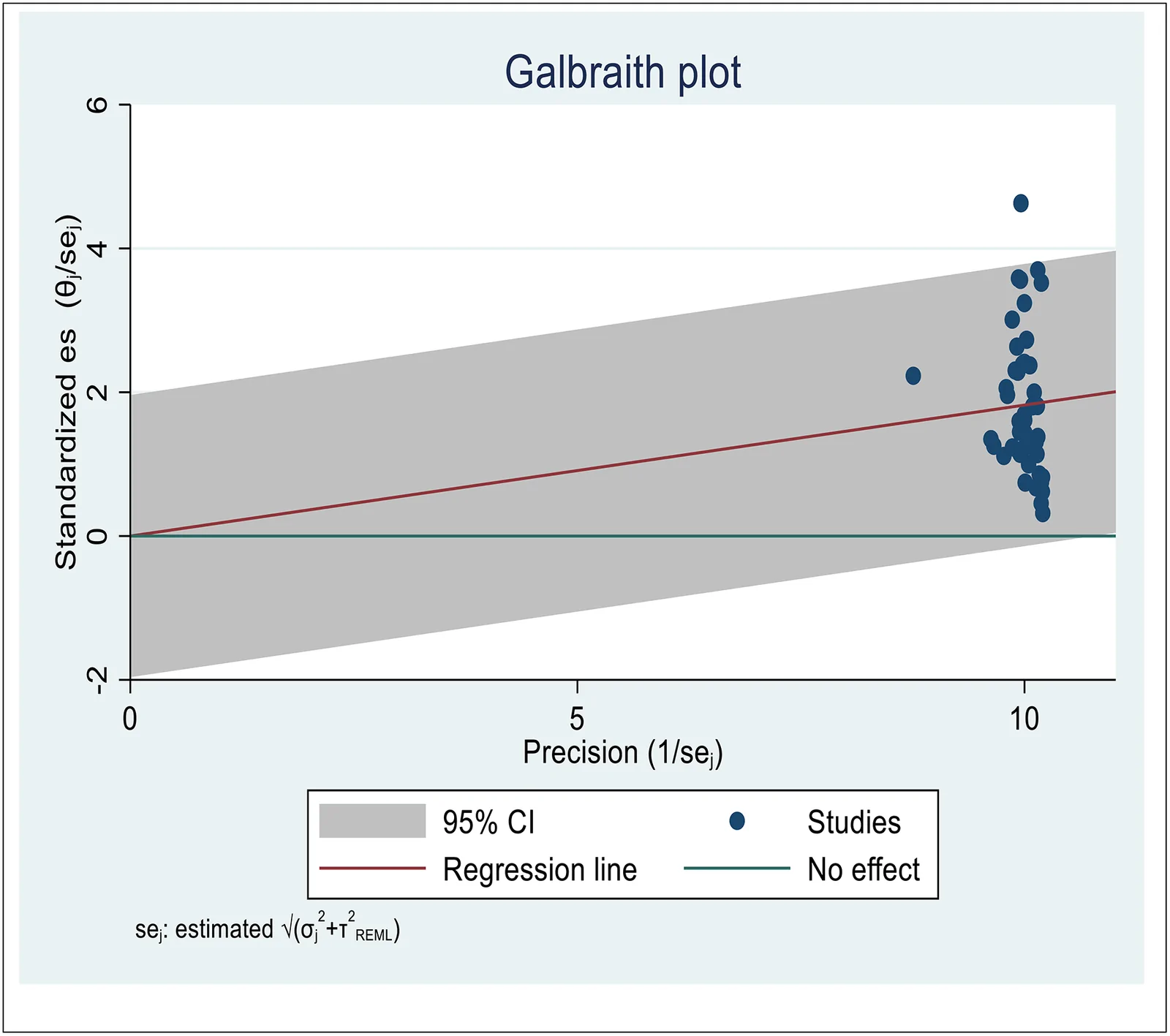
Galbraith plot for the pooled prevalence of norovirus and rotavirus among under five children with diarrheal infection in LMICs.

Subgroup analysis based on outcome showed that the pooled prevalence of norovirus and rotavirus was 13% (95% CI: 11–15) and 24% (95% CI: 19.6–28.3), respectively (Fig 5). Regarding subgroup analysis by region, the prevalence of norovirus and rotavirus was highest in Southern Africa at 26% (95% CI: 8–45), followed by North Africa at 21% (95% CI: 11–31), South Asia at 19% (95% CI: 15–24), Central Africa at 16% (95% CI: 1–30), West Africa at 16% (95% CI: 8–23), East Africa at 14% (95% CI: 11–17), Central America at 13% (95% CI: 9–18), and the Middle East at 11% (95% CI: 9–14) (Fig 6). The pooled prevalence of norovirus and rotavirus according to study design was 17% (95% CI: 14–20) for cross-sectional studies and 21% (95% CI: 16–27) for surveillance studies (Fig 7). Regarding subgroup analysis by detection method, the pooled prevalence was 25% (95% CI: 20–29) for ELISA combined with RT-PCR, 18% (95% CI: 9–27) for ELISA combined with multiplex PCR, and 13% (95% CI: 10–16) for RT-PCR alone. Meta-regression analysis indicated that sample size was not significantly associated with the effect size (β = −1.98 × 10^−6^, p = 0.887), despite substantial heterogeneity (I² = 98.72%, Q-res p < 0.001).

**Fig 5:**
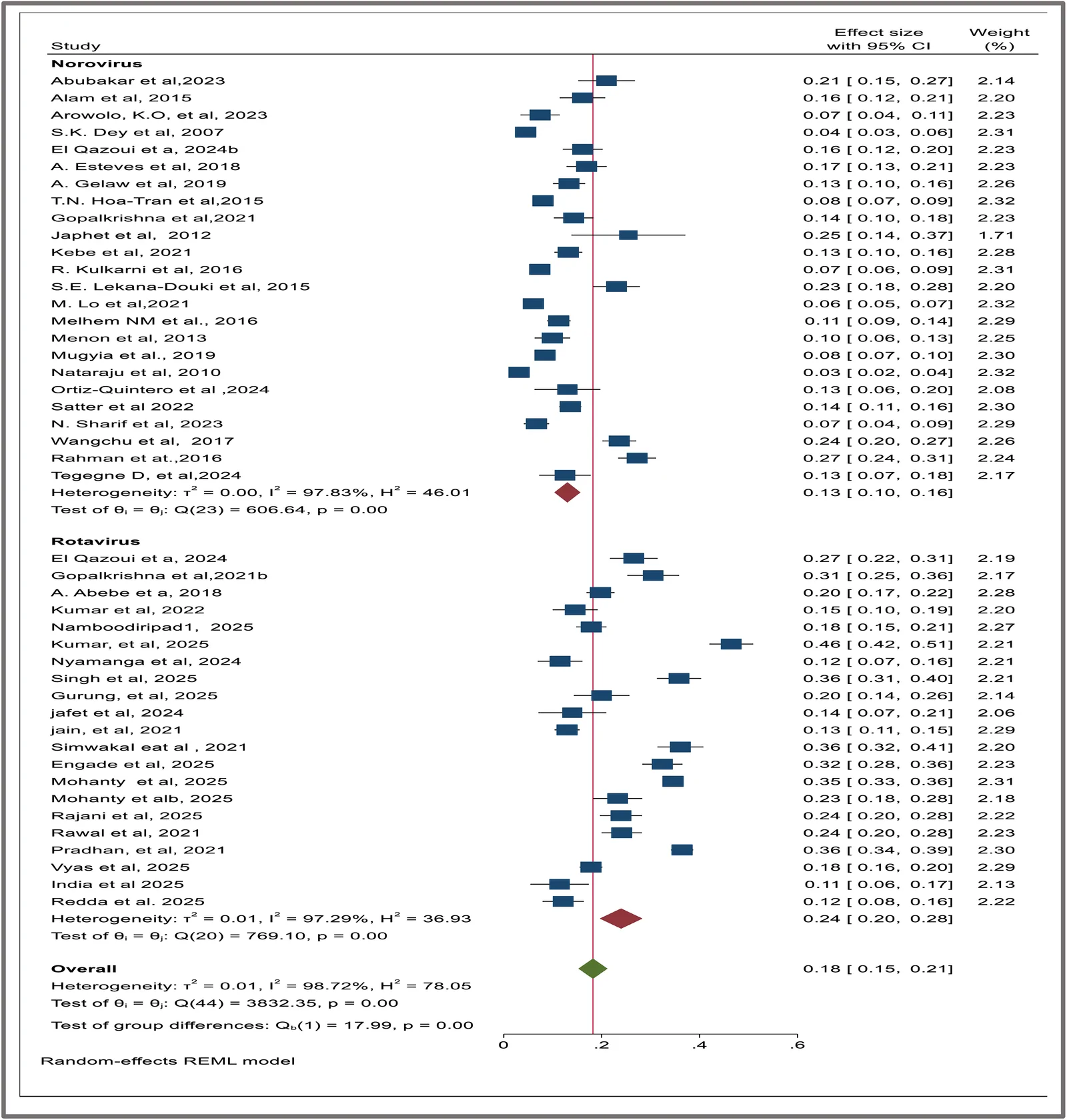
Forest plot showing the subgroup analysis by outcome variable for the pooled prevalence of norovirus and rotavirus among under five children with diarrheal infection in LMICs.

**Fig 6:**
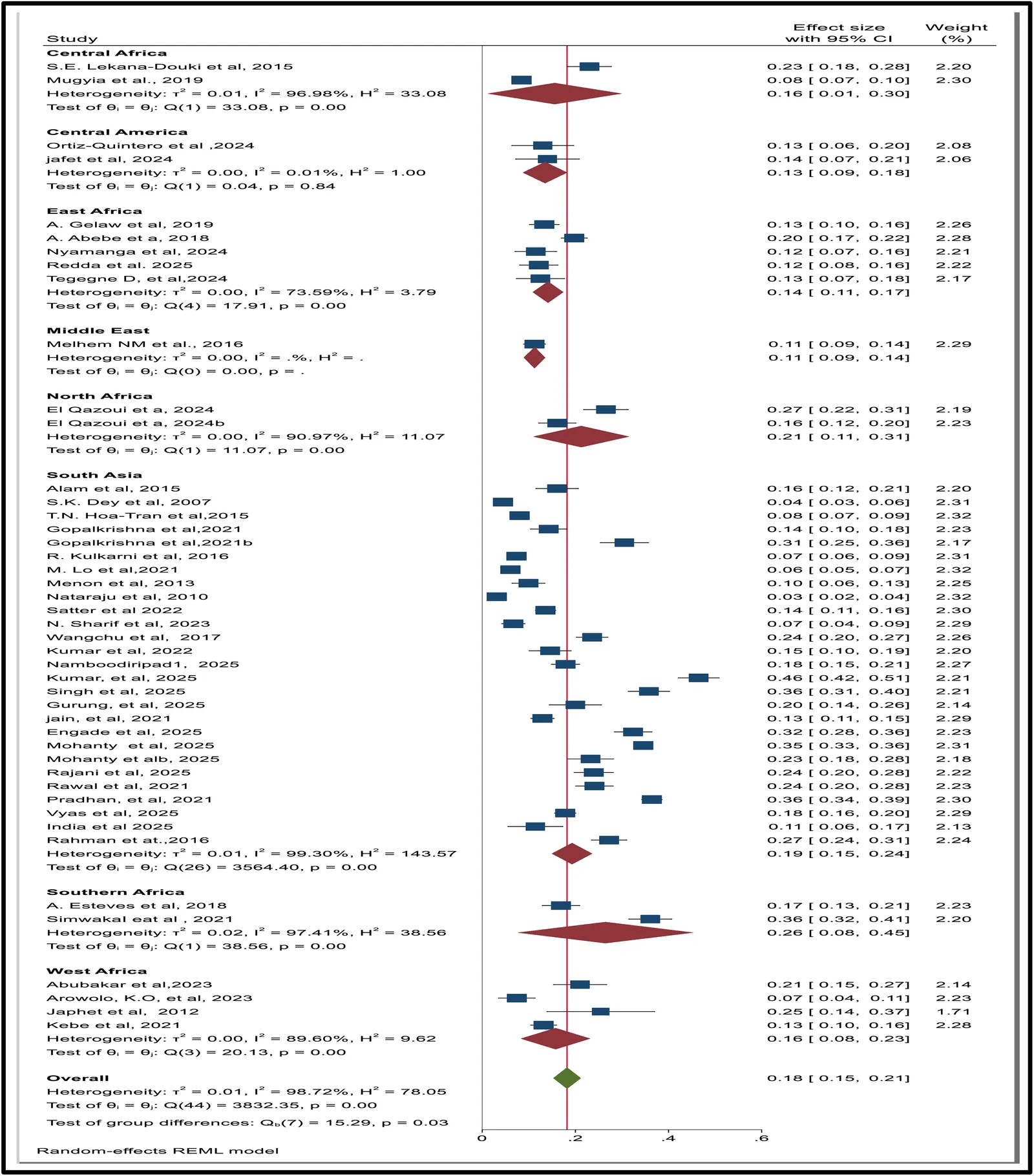
Forest plot showing the subgroup analysis by region for the pooled prevalence of norovirus and rotavirus among under five children with diarrheal infection in LMICs.

**Fig 7:**
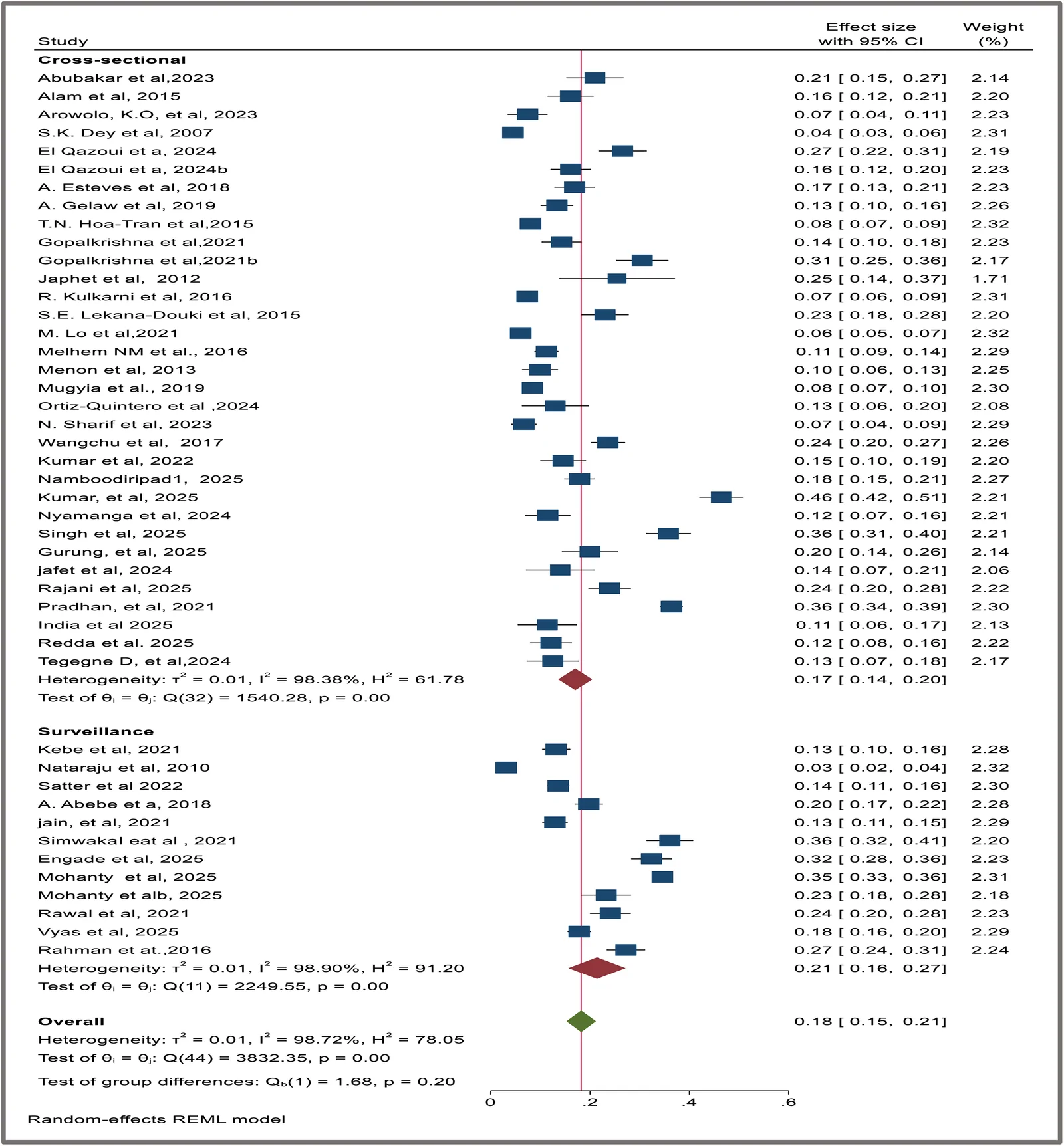
Forest plot showing the subgroup analysis by study design for the pooled prevalence of norovirus and rotavirus among under five children with diarrheal infection in LMICs.

The leave-one-out meta-analysis was conducted to evaluate the influence of individual studies on the overall effect size (Fig 8). The results showed that no single study had a significant impact on the pooled estimate, as the overall effect remained stable when each study was sequentially omitted, with the p-values remaining significant. Egger’s regression test indicated significant small-study effects (β = 3.25, p = 0.035). Similarly, Begg’s rank correlation test provided evidence of publication bias (p = 0.039). These findings suggest the presence of potential publication bias among the included studies (Fig 9). Additionally, a trim-and-fill analysis was performed, which showed that the overall pooled estimate remained unchanged despite evidence of possible publication bias.

**Fig 8:**
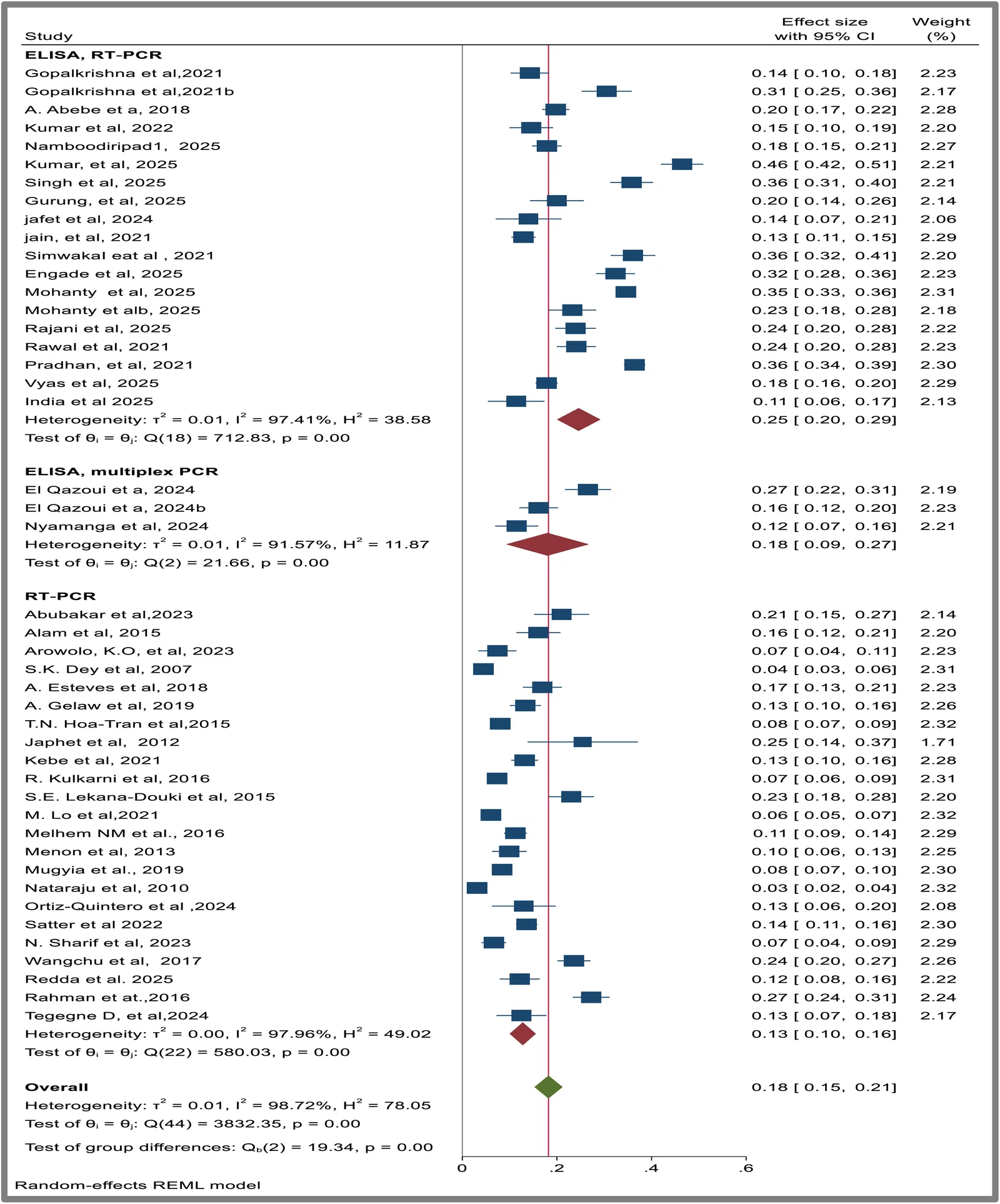
Forest plot showing the subgroup analysis by detection methods for the pooled prevalence of norovirus and rotavirus among under five children with diarrheal infection in LMICs.

**Fig 9:**
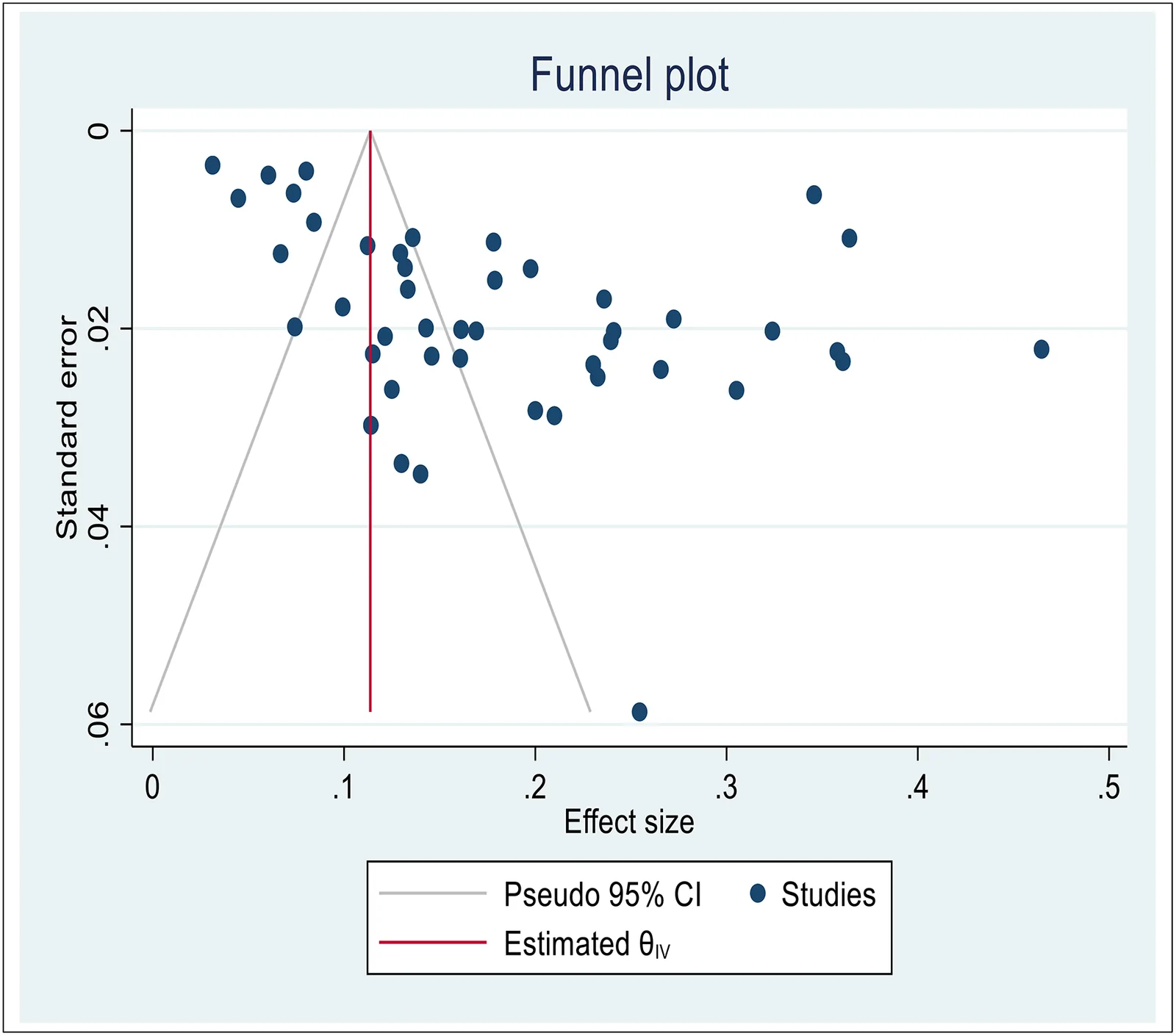
Funnel plot for the pooled prevalence of norovirus and rotavirus among under five children with diarrheal infection in LMICs.

### Genotype distribution *Norovirus* and *Rotavirus*

A total of 43 studies were included to synthesize evidence on the genotype distribution of norovirus and rotavirus. A total of 35,704 samples were collected from under five children, 9,889 were sequenced to determine genotype, yielding 1,840 identified genotypes. Most norovirus detections belonged to Genogroup II (GII), 665 of GII-positive cases were reported across Nigeria, Pakistan, Morocco, Senegal, Gabon, Lebanon, Cameroon, India, Honduras, Bangladesh, and Ethiopia [25,40,43,45,49,50,53,54,56–58,60,62,63]. Genogroup I (GI) accounted for 129 cases reported from Nigeria, Morocco, Angola, India, Senegal, Gabon, Lebanon, Cameroon, Honduras, and Bhutan [23,25,41,50,53–58,60,63]. Sub genotype analysis showed that GII.4 was the most common GII subtype, with 563 cases reported from Bangladesh, Angola, Ethiopia, Nepal, Senegal, Lebanon, India, Cameroon, and Bhutan [23,24,40–44,46,55–57,60,61,63]. The next most frequent subtype was GII.3, with 145 cases reported from several LMICs including Angola, Nepal, Lebanon, India, Bangladesh, and Bhutan [24,40,41,43,55,56,60,61](Fig 10).

**Fig 10:**
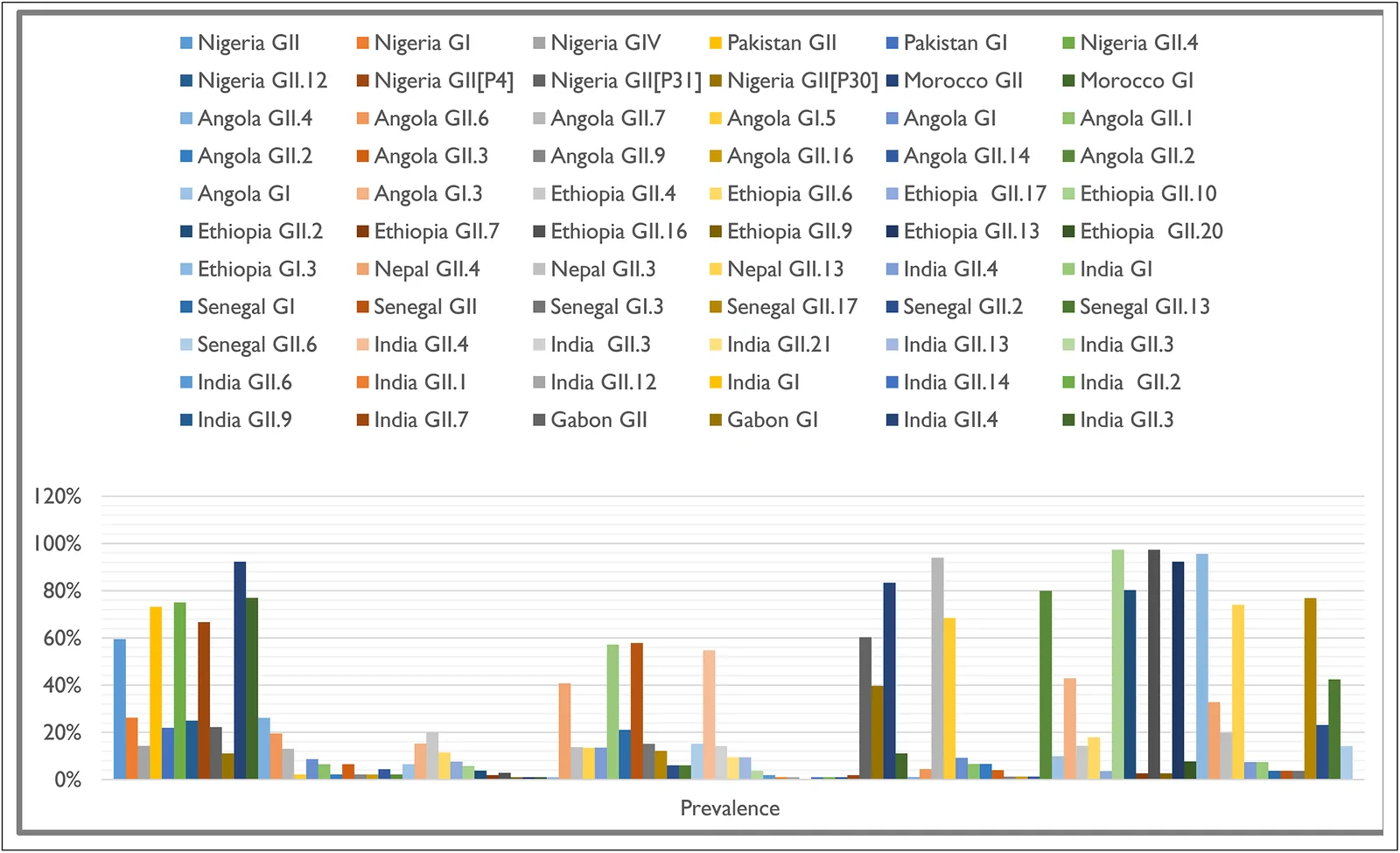
Distribution of Norovirus Genotype among children under five with diarrheal infection in lower-middle-income country.

Across low- and middle-income countries (LMICs) after rotavirus vaccine introduction, the most frequently observed genotype was G3[P8], with 1,437 detections reported primarily from India, and also from Ethiopia and Kenya [26–28,30–35,37,39,47,48,59]. The second most common genotype was G1[P8], with 501 reported detections from Morocco, Ethiopia, and India [23,26–31,33,35,36,39,47,54,64]. The third most frequent was G2[P4], accounting for 489 detections reported in India, Ethiopia, Honduras, and Zambia [23,26–36,47,52,64]. A total of 384 mixed-type rotavirus detections were reported, mainly from studies in India and Zambia, with the majority coming from India [26,27,30,31,33,35,38,39,64]. The review also recorded five partially typable detections from India [30] and 16 non-typable detections from Ethiopia(Fig 11).

**Fig 11:**
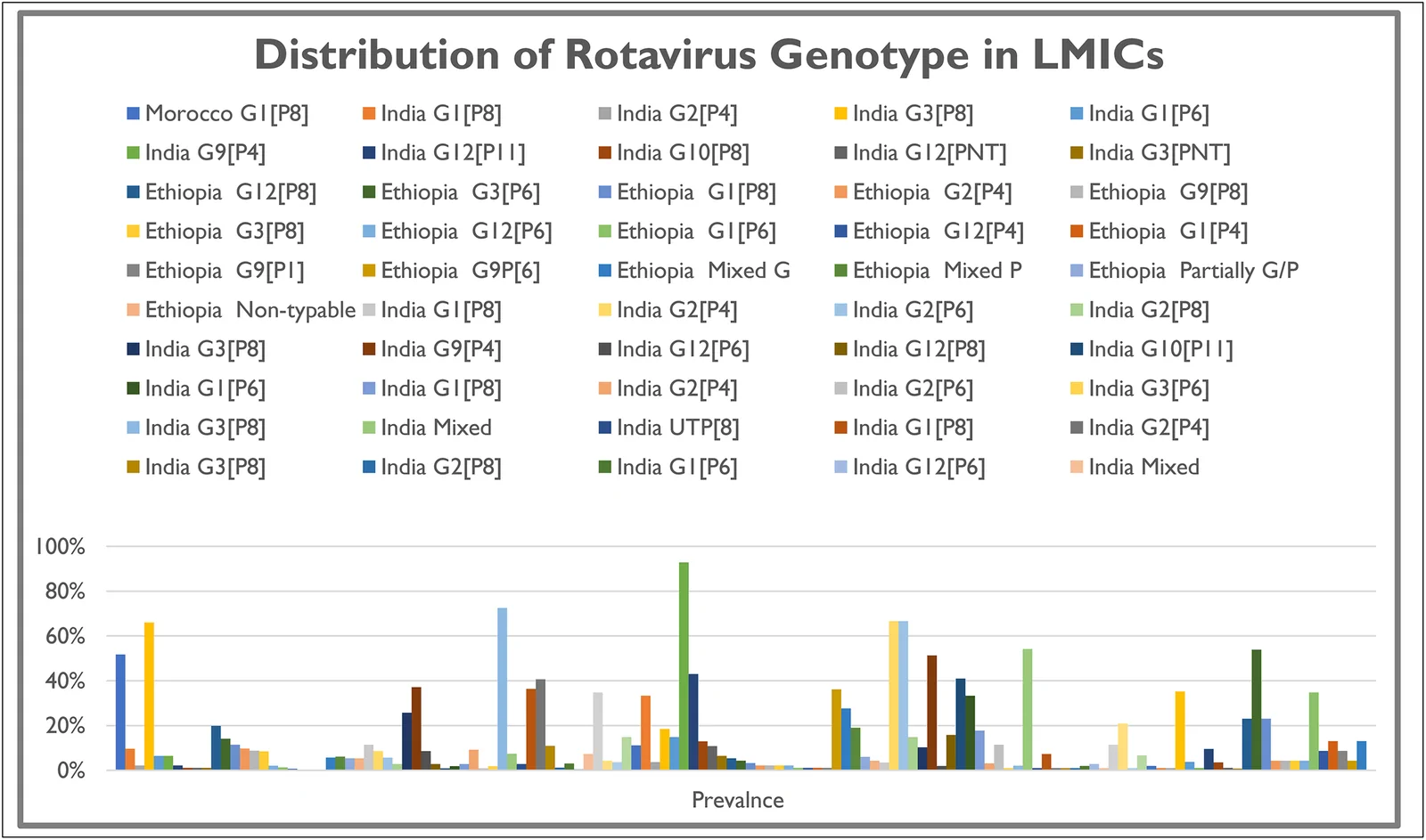
Distribution of Rotavirus Genotype among children under five with diarrheal infection in lower-middle-income countries.

### Pooled prevalence of predominant genotype *Norovirus* and *Rotavirus*

The predominant norovirus genotype was genogroup II (GII), with a pooled prevalence of 76% (95% CI: 61–91%), while the GI genogroup had a pooled prevalence of 23% (95% CI: 9–37%). Within GII, the sub-genotype GII.4 accounted for 53% (95% CI: 39–68%) of cases. Regarding rotavirus genotypes, G3P[8] was the most prevalent, with an overall pooled prevalence of 39% (95% CI: 28–51%), followed by G1P[8] with a pooled prevalence of 17% (95% CI: 10–25%). The pooled prevalence estimates for GII, GII.4, GI, G3P[8], and G1P[8] are presented in Figs 12–16, respectively.

**Fig 12:**
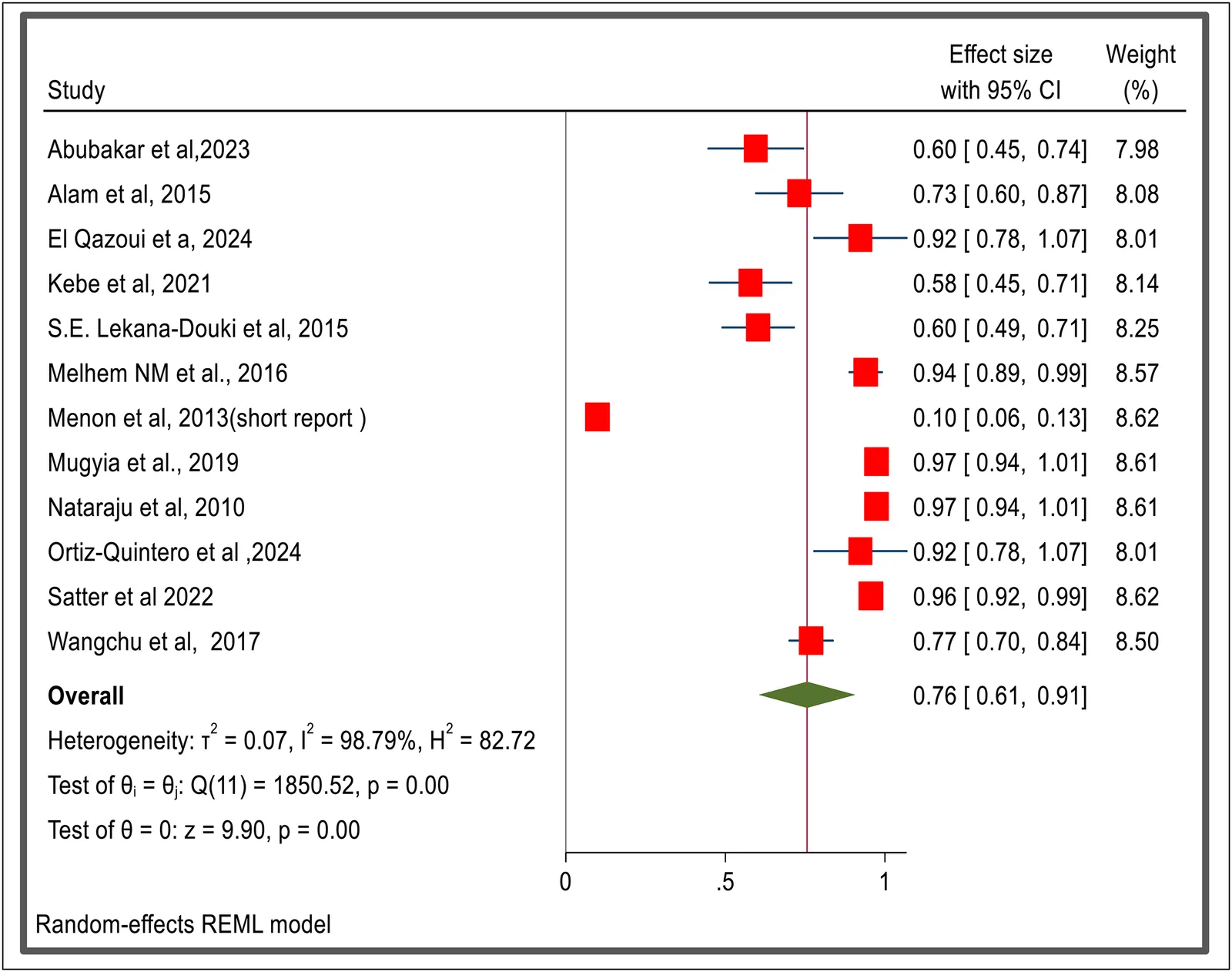
Pooled prevalence of GII among children under five with diarrheal infection in lower-middle-income countries.

**Fig 13:**
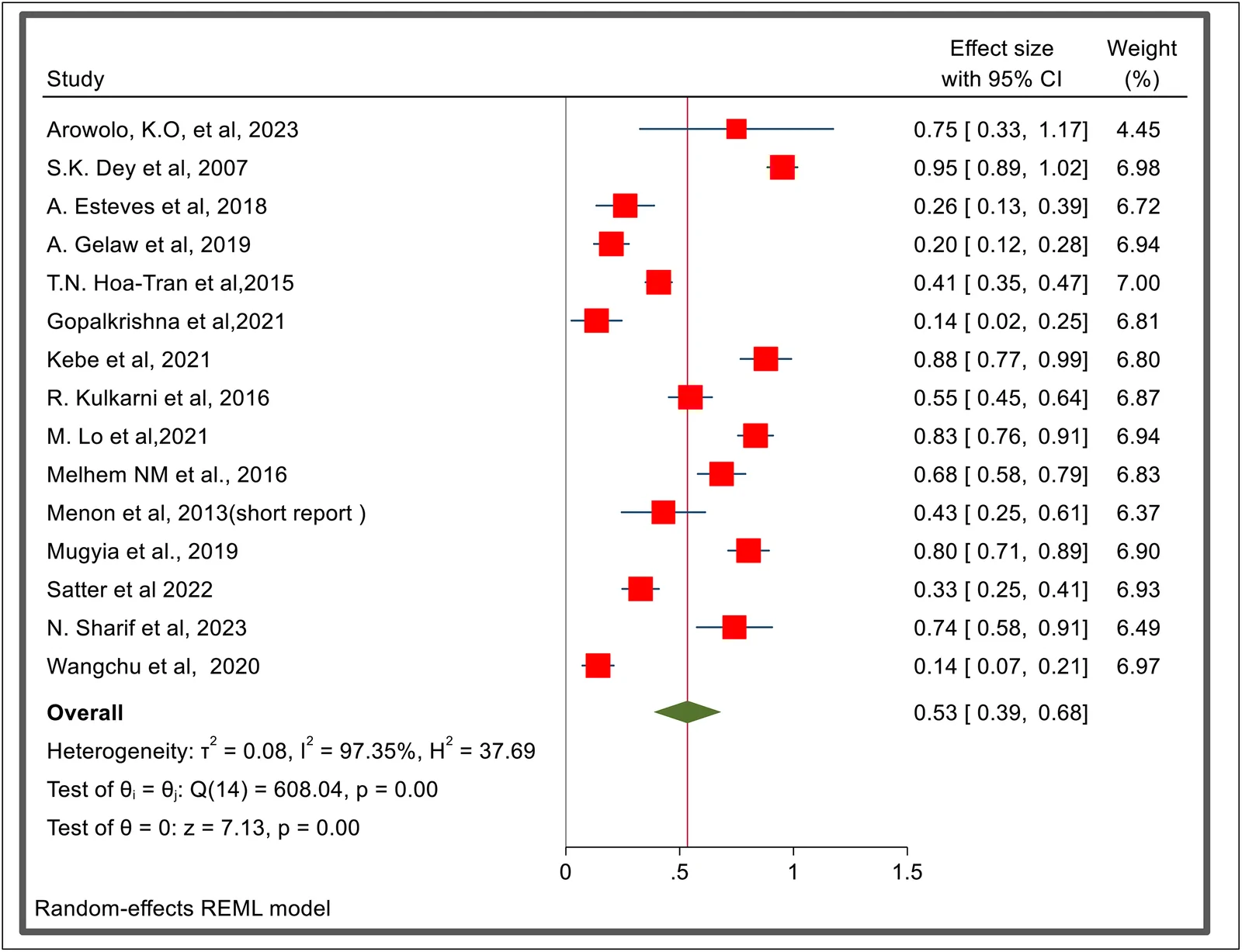
Pooled prevalence of GII.4 among children under five with diarrheal infection in lower-middle-income countries.

**Fig 14:**
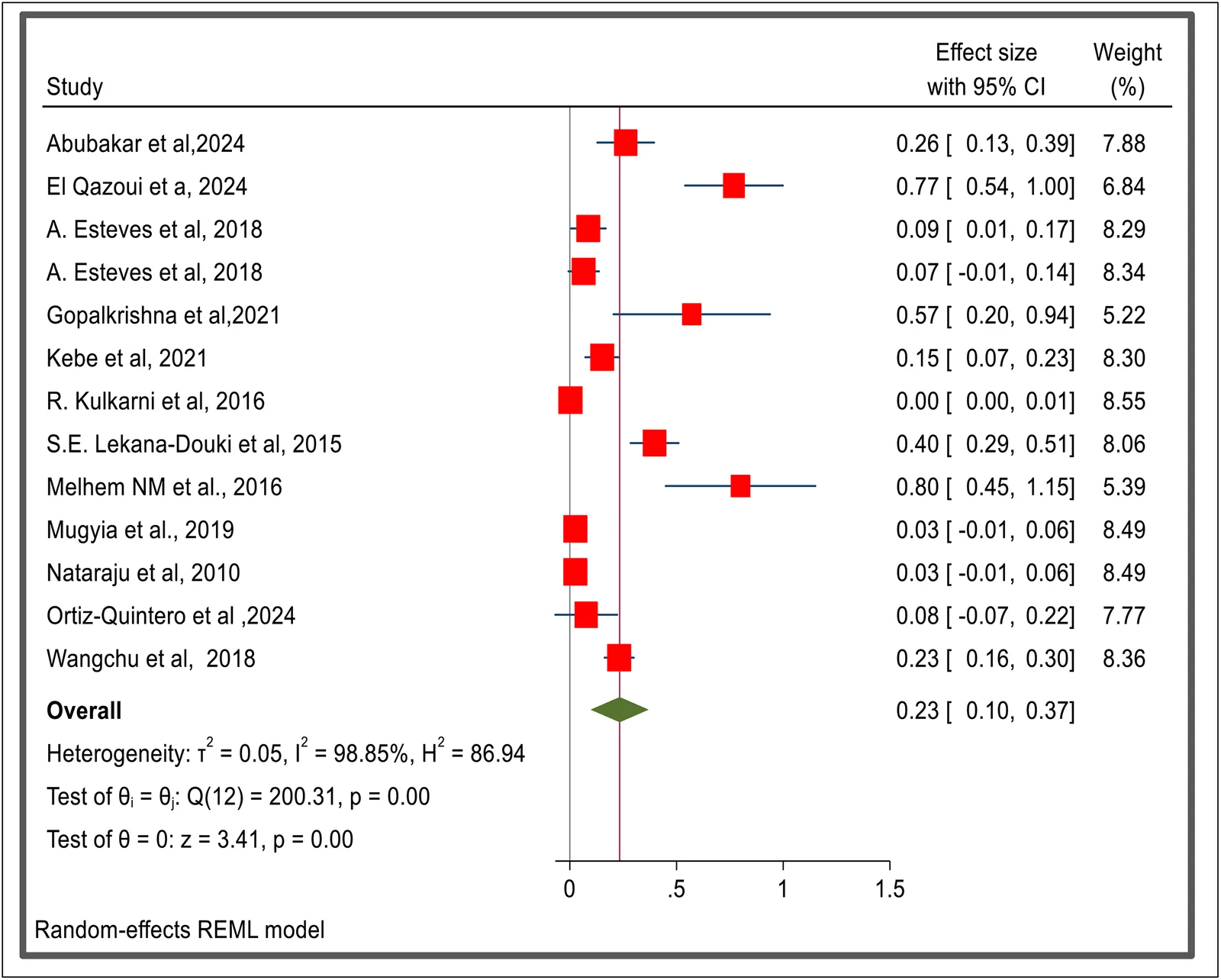
Pooled prevalence of GI among children under five with diarrheal infection in lower-middle-income countries.

**Fig 15:**
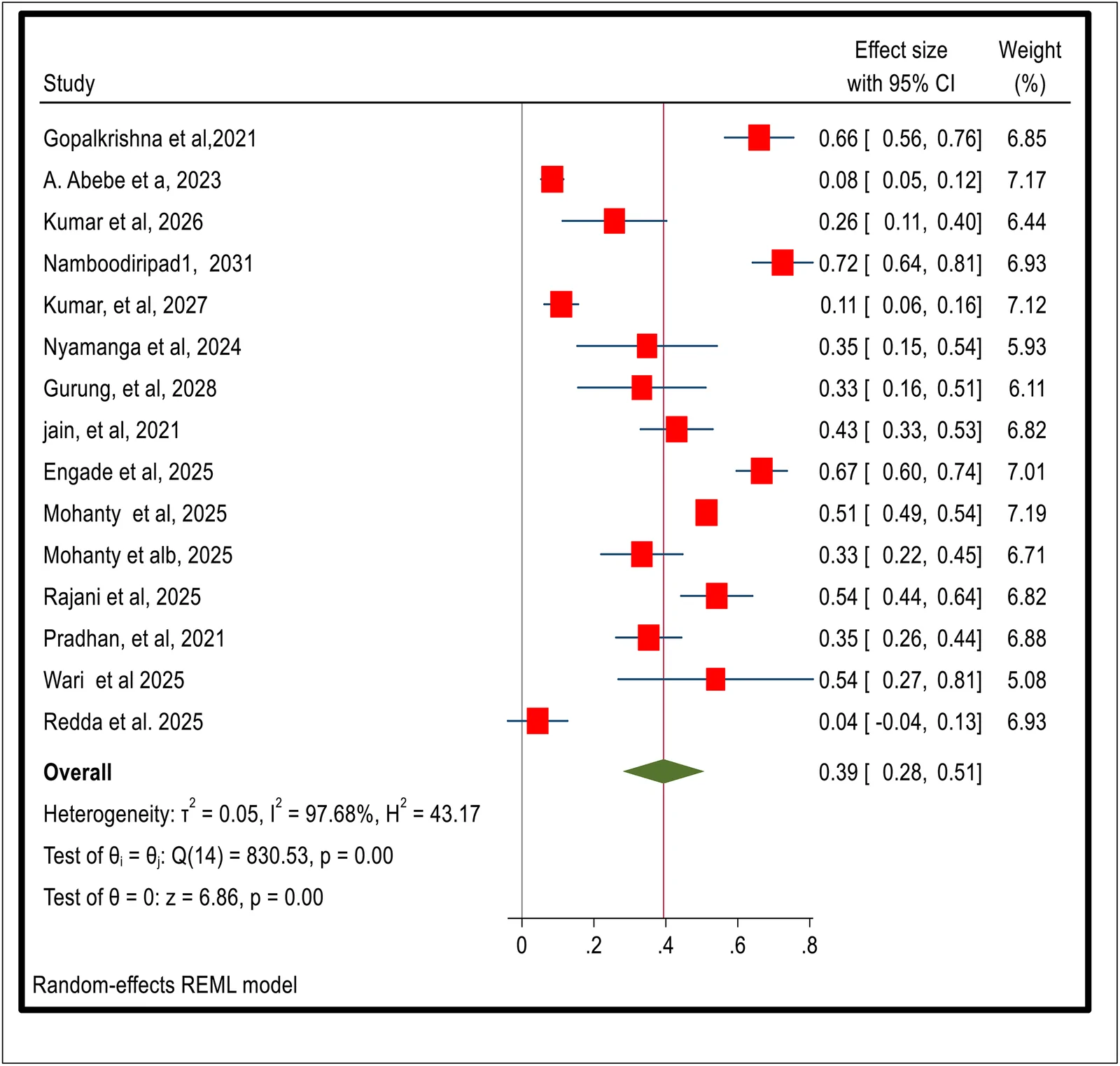
Pooled prevalence of G3P [8] among children under five with diarrheal infection in lower-middle-income countries.

**Fig 16:**
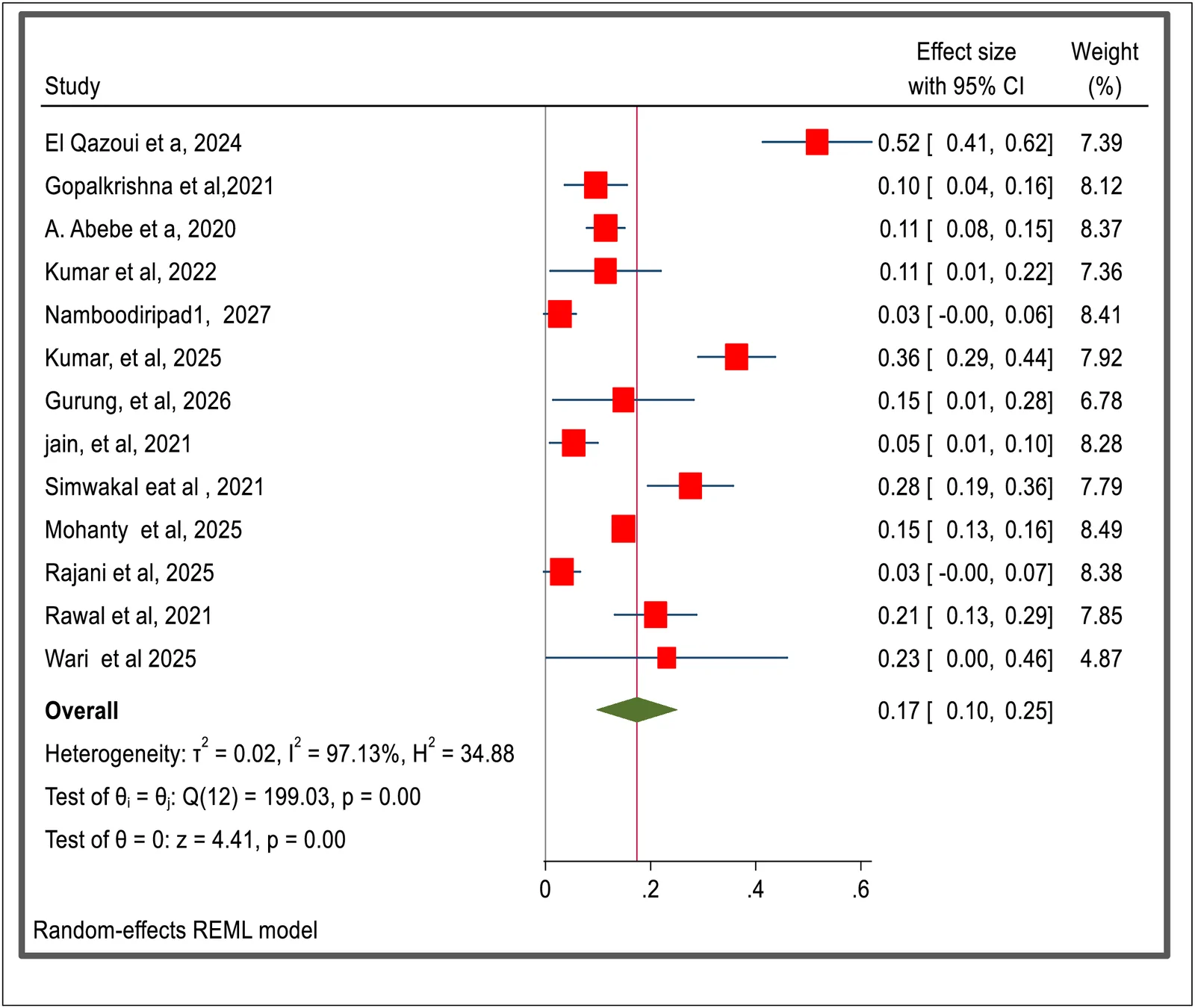
Pooled prevalence of G1P[8] among children under five with diarrheal infection in lower-middle-income countries.

## Discussion

Despite technological advances, improved treatments, widespread vaccine use, and overall better sanitation, viral diarrhea remains a leading cause of childhood mortality in low- and middle-income countries. Transmission often arises periodically due to scarce resources, high population density, and low public awareness of how the infection spread in these counties [65–67]. Therefore, knowing the burden, prevalence of norovirus and rotavirus, it also equally important to knowing genotype distribution for vaccine targeting and development [68]. Therefore, this systematic review and meta-analysis ultimately included 45 studies from low- and middle-income countries (LMICs) reporting the prevalence of norovirus and rotavirus, and 43 studies reporting genotype distribution, among children under five years of age with diarrheal infection following the introduction of the rotavirus vaccine.

The overall pooled prevalence of norovirus and rotavirus was 18% (95% CI: 15%–21%). This result highlight that both viruses remain significant causes of acute gastroenteritis In LMICs and nearly one in five diarrheal cases may be associated with enteric viral infections (Norovirus or Rotavirus). Subgroup analysis based on outcome revealed that the pooled prevalence of norovirus was 13% (95% CI: 11%–15%). This finding is consistent with previously reported pooled estimates from China (11.54) [69], Latin America 15% [70], and Africa 15% [70]. However, the present estimate is lower than findings from global systematic reviews and meta-analyses, which reported prevalence of 17.7% [71], and 18% [72], respectively. In contrast, the current finding is higher than the pooled prevalence reported in sub-Saharan Africa (6.6%) [73]. Additionally, previous studies in Africa have reported pooled norovirus prevalence of 15% (95% CI: 15%–18%) in the pre rotavirus vaccination period and 13% (95% CI: 9%–17%) in the post-vaccination period [74]. The observed discrepancies may be due to differences in the sensitivity of detection methods [75], variations in sanitation, hygiene practices [76], and access to healthcare, as well as differences in sample size, including hospital-based cross-sectional studies versus larger surveillance studies [77]. Temporal factors, including the introduction and scale-up of rotavirus vaccination programs, may also influence the observed burden of norovirus by altering pathogen dynamics.

In the current systematic review and meta-analysis, the pooled prevalence rotavirus found to be 24% (95% CI: 19.6%–28.3%). This means out of 4 children with diarrheal infection one may be due to rotavirus infection. This estimate is comparable to findings reported from Burkina Faso (27.7%) [78], South Africa (24%) [79], and Ethiopia (23%) [80], supporting the evidence that rotavirus remains a common cause of acute diarrheal disease in sub-Saharan Africa. However, the current estimate is lower than pooled prevalence reports from Africa overall (31.0%) [81], Bangladesh (30.1%) [82], and sub-Saharan Africa (29.2%) [73]. In contrast, the present finding is higher than estimates reported from Latin America (16.1%) following the introduction of rotavirus vaccination [83]. Notably, studies from this region have documented a decline in rotavirus prevalence from 24.3% to 16.1% after vaccine implementation, highlighting the substantial impact of vaccination programs in reducing disease burden. These variations may be explained by differences in rotavirus vaccine introduction year, vaccine coverage across regions [84], seasonal variation, geographic differences, and health system strength are also contributing factors to the observed differences in detection reports.

In the current systematic review and meta-analysis, the most frequently detected norovirus genotype was GII, with a pooled prevalence of 76% (95% CI: 61%–91%). This estimate is comparable to the global pooled prevalence of 84.6% [81].This genotype is considered the globally dominant strain in norovirus infections. However, the estimate is slightly lower than the systematic review and meta-analysis conducted in Africa (91.2%) [74]. At the sub-genotype level, GII.4 was the most prevalent, with a pooled estimate of 53% (95% CI: 39%–68%). This finding is consistent with reports from China (45.18%) and global estimates (59.3%) [71], which confirm GII.4 as the most common norovirus genotype circulating in the population. However, the observed prevalence is lower than the systematic review and meta-analysis reported from Africa (79.3%) [81]. In addition to GII and GII.4, other genotypes such as GII.3 were also reported, with prevalence estimates of 11.92% in China [69] and 14.9% globally [71], suggesting moderate but consistent circulation across regions.

In the current systematic review and meta-analysis, the most common G/P genotype combination of rotavirus was G3P[8], with a pooled prevalence of 39% (95% CI: 28%–51%). This estimate is higher than reports from Asia (24%) [10], where G3P[8] has also been identified as a predominant circulating genotype. In contrast, lower prevalence estimates have been reported from Africa (11.7%) [81], Ethiopia (12.9%) [80], and South Africa (7.02%) [79], suggesting considerable regional variation in genotype distribution. Similarly, G1P[8] was identified as the second most common genotype in the current systematic review and meta-analysis, with a pooled prevalence of 17% (95% CI: 10%–25%). This estimate is comparable to findings from South Africa and Nicaragua (20.3%) [83]. However, it is higher than estimates reported from Asia (8%) [13], Africa (7.1%) [81], and Chile (6.8%) [83], while remaining lower than reports from several Latin American countries, including Brazil (54.2%), Argentina (46.6%), Ecuador (50.0%), and Colombia (57.3%) [83]. Therefore, rotavirus remains the major public health problem in LMICs even after the introduction of rotavirus vaccine. It is very important to strengthen immunization programs, improve vaccine accessibility and enhance surveillance systems to monitor disease trends and vaccine impact.

### Limitations of the study

The findings of this study may inform evidence-based decision-making; however, several limitations should be considered. First, the search was restricted to studies published in English, which may have introduced language bias by excluding potentially relevant studies published in other languages. This restriction may have reduced the comprehensiveness and geographical representativeness of the evidence included in the review. Second, the majority of the included studies were cross-sectional, which limits the ability to establish temporal associations. Third, substantial heterogeneity was observed across studies, which may limit comparability and should be considered when interpreting the pooled estimates. Fourth, the possibility of publication bias may have affected the pooled estimates, as studies with statistically significant or positive findings may be more likely to be published than studies with null or negative findings. Finally, the absence or limited availability of studies from some LMIC regions reduces the generalizability of the findings, while the large proportion of studies from India may have disproportionately influenced the overall pooled estimates and limited comparability across geographical regions.

## Conclusions and recommendations

### Conclusions

This systematic review and meta-analysis synthesizes evidence. on the prevalence and genotype distribution of norovirus and rotavirus among children under five years of age in low- and middle-income countries following rotavirus vaccine introduction. Both viruses remain major causes of diarrheal disease in this population. Approximately one in five children with diarrheal illness may be attributable to at least one of these viruses. Specifically, norovirus was associated with approximately one in eight diarrheal cases, while rotavirus was associated with approximately one in four. Among norovirus genotypes, GII particularly the GII.4 subgenotype was predominant, whereas G3[P8] and G1[P8] were the most commonly identified rotavirus genotypes. However, given the considerable heterogeneity in reported prevalence and genotype distribution across studies, these findings should not be considered conclusive.

### Recommendations

Policymakers, programmers and researchers should work closely together to regular monitoring and strengthening surveillance systems to track the prevalence, genotype shifts, and genotype distribution within the population to control and reduce the infection. Vaccine manufacturers should consider the full range of circulating genotypes prior to vaccine manufacture, and the vaccine should include at least the predominant rotavirus genotypes associated with infection in low- and middle-income countries. Health interventions, as well as water, sanitation, and hygiene (WASH) practices, should be strengthened. Future research should focus on longitudinal and large-scale surveillance studies using molecular methods.

## Supporting information

S1 Table Full search strategy for a systematic review and meta-analysis for prevalence and genotype distribution of norovirus and rotavirus among children under five with acute diarrhea in lower-middle-income countries after rotavirus vaccine introduction from February 20–28/2026. (DOCX)

S2 Table List of LMICs with the year of rotavirus vaccine introduction for prevalence and genotype distribution of norovirus and rotavirus among children under five with acute diarrhea in lower-middle-income countries after rotavirus vaccine introduction, 2026. (DOCX)

S3 Table PRISMA 2020 checklist. (DOCX)

S4 Table Risk of bias and quality assessment results of the 45 studies included in the systematic review for prevalence and genotype distribution of norovirus and rotavirus among children under five with acute diarrhea in lower-middle-income countries after rotavirus vaccine introduction, 2026. (DOCX)
