# Supplementary material for: Prevalence and genotype distribution of norovirus and rotavirus among children under five with diarrheal infection in lower-middle-income countries after Rotavirus vaccine introduction: A systematic review and meta-analysis
Source: PLoS One. 2026 Oct 13;21(10):e0359886. doi: 10.1371/journal.pone.0359886 (PMC13660929; doi:10.1371/journal.pone.0359886)
Supplement: Supplementary file 1 [file pone.0359886.s005.pdf]

Peer Review Report

Manuscript: Prevalence and Genotype Distribution of Norovirus and Rotavirus Among Children Under Five with Diarrheal Infection in Lower- and Middle-Income Countries After Rotavirus Vaccine Introduction A Systematic Review and Meta-Analysis

Type of submission: Systematic Review and Meta-Analysis

Recommendation: Accept with Revisions

1. General Introduction & Summary

Herein, the authors present a systematic review and meta-analysis of the pooled prevalence and genotype distribution of norovirus and rotavirus among children under five with diarrheal infection across 45 studies (35,704 children) from low- and middle-income countries (LMICs), restricted to the period following national rotavirus vaccine introduction. Using a random-effects (REML) model, the overall pooled prevalence was 18% (95% CI: 15–21%), with rotavirus (24%) more prevalent than norovirus (13%). Heterogeneity was substantial throughout ($I^2 \approx$ 98–99%) and was explored via subgroup and meta-regression analyses. GII, particularly GII.4, was the dominant norovirus genotype, while G3P[8] and G1[P8] led among rotavirus genotypes. The authors conclude that both viruses remain a significant burden in LMICs post-vaccination and call for continued genomic surveillance.

2. Academic Contribution, Strengths, and Weaknesses

Strengths. This is a timely and well-executed contribution. Notably, restricting rotavirus inclusion to the post-vaccine period isolates a genuinely contemporary epidemiological picture, and combining both pathogens (rather than reviewing them separately, as most prior work does) offers a fuller picture directly relevant to multivalent vaccine design. The methodology is rigorous PROSPERO registration, independent dual screening/extraction, JBI quality appraisal, and a comprehensive heterogeneity/bias toolkit (Q-statistic, I^2, Galbraith and funnel plots, Egger's/Begg's tests, leave-one-out sensitivity analysis, trim-and-fill, meta-regression) and the 17-country geographic spread supports broad relevance across the LMIC context. The authors' conclusions are also appropriately hedged given the very high heterogeneity, which reflects good scientific practice.

Weaknesses. Several numerical inconsistencies recur across sections and should be reconciled: the overall I^2 is variously reported as 98.85%, 98.72%, and 98.67%; GII pooled prevalence appears as both 76% and 75%; the GI prevalence (23%) is paired with a CI (10–17%) that does not contain the point estimate (Figure 14 gives 10–37%); and the stated rotavirus prevalence range ("17.90–24.10%") does not match several Table 1 entries as high as 46.50%. Figure citations in the sensitivity-analysis subsection also appear mismatched against the actual figure captions, and a citation string in the genotype section is garbled, likely from a page-break artifact.

Beyond these, a more substantive point: given I^2 near 98–99% throughout, a single pooled headline figure (18%) may understate how disparate the underlying data are, even with subgroup analysis performed. Notably, the manuscript's claim that "one study reported both viruses" appears incorrect Table 1 shows two (El Qazoui et al., 2024 and Gopalkrishna et al., 2021, each listed under separate norovirus/rotavirus rows). We would also flag that nearly half of included studies (20/45) originate from India, which limits generalizability to other LMIC regions; that the English-only search restriction may have compounded the detected publication bias by excluding French/Spanish/Portuguese-language literature; and that pooling cross-sectional and surveillance designs together, despite differing methodologies, risks obscuring real differences in burden. Finally, if the Data Availability Statement restricts data to "available upon request," this would not meet PLOS ONE's minimal-dataset policy we could not verify this wording against the material provided and would ask the editor to confirm with the authors.

None of these issues undermine the manuscript's core validity or contribution; they are correctable through text and data reconciliation, consistent with Accept with Text Revisions. However, several of the items above are substantive data-accuracy checks, not merely stylistic ones, and should be resolved before production.

---

3. Line-by-Line Revisions & Language Corrections

*Representative **examples** are given for each manuscript section; this is not an exhaustive list. Citation-bracket spacing (e.g., "word[12]" instead of "word [12]") recurs dozens of times throughout and should be corrected globally.*

Abstract

Line	Original Text	Suggested Revision	Justification
42	"children presenting with diarrheal illness infected with at least one of these viruses"	"…illness were infected with at least one of these viruses"	Missing auxiliary verb.
45–46	"monitor genotype shifts among population"	"monitor genotype shifts within the population"	Missing article/wrong preposition.

Introduction

Line	Original Text	Suggested Revision	Justification
64	"Norovirus (NoV) is belongs to the Caliciviridae family…single stranded, no enveloped"	"Norovirus (NoV) belongs to the Caliciviridae family…single-stranded, non-enveloped"	Double verb; incorrect terminology/hyphenation.
78	"especially on under five children's"	"especially on children under five"	Misplaced apostrophe, wrong word order.

Line	Original Text	Suggested Revision	Justification
87	"to the best of my knowledge"	"to the best of our knowledge"	Multi-author manuscript should use "our."

Methods

Line	Original Text	Suggested Revision	Justification
126–127	"the search result were imported into Rayyan(AI-Powered…"	"the search results were imported into Rayyan (AI-Powered…"	Subject–verb agreement; missing space.
140–141	"The overall quality of each eligible article were evaluated"	"…was evaluated"	Subject–verb agreement.

Results

Line	Original Text	Suggested Revision	Justification
164–165	"Finally,45 articles were included…"	"Finally, 45 articles were included…"	Missing space after comma.
190–191	"…one study reported both viruses"	"…two studies reported both viruses"	Factual error Table 1 shows El Qazoui et al. 2024 and Gopalkrishna et al. 2021 each reporting both viruses.
266–267	GI prevalence "23% (95% CI: 10–17%)"	"23% (95% CI: 10–37%)" *(verify)*	CI does not contain the point estimate; inconsistent with Figure 14.

Discussion

Line	Original Text	Suggested Revision	Justification
278–280	"Transmission often a rises periodically…how the infection spread in these counties"	"…often arises periodically…how infection spreads in these countries"	Typos; subject–verb agreement.
320–322	"…84.6% [81], his genotype is considered…"	"…84.6% [81]. This genotype is considered…"	Typo ("his" for "This"); comma splice.

Conclusions, Recommendations & References

Line	Original Text	Suggested Revision	Justification
356–357	"This This systematic review and meta-analysis synthesize evidence…"	"This systematic review and meta-analysis synthesizes evidence…"	Duplicated word; subject–verb agreement.
Ref 64	"Children s Hospital in 2016, in Lusaka Zambia"	"Children's Hospital in 2016, in Lusaka, Zambia"	Missing apostrophe and comma.

Line	Original Text	Suggested Revision	Justification
Table 1, Row 34	"SimwakaI eat al , 2021"	"Simwaka et al., 2021"	Typo in author name and "et al."

Summary for the Editor

This is a methodologically sound and topically important review, recommended for **Accept with Revisions**. Most fixes are linguistic and typographical, but we ask the authors to specifically reconcile the internal numerical inconsistencies (I^2 values, GII prevalence, GI confidence interval, the "one vs. two studies" discrepancy, and mismatched figure citations) and to address the India-heavy geographic weighting, the English-language restriction's possible link to publication bias, and the Data Availability Statement's compliance with PLOS ONE policy, before the manuscript proceeds to production.
