# Supplementary material for: Prevalence and genotype distribution of norovirus and rotavirus among children under five with diarrheal infection in lower-middle-income countries after Rotavirus vaccine introduction: A systematic review and meta-analysis
Source: PLoS One. 2026 Oct 13;21(10):e0359886. doi: 10.1371/journal.pone.0359886 (PMC13660929; doi:10.1371/journal.pone.0359886)
Supplement: Supplementary file 2 [file pone.0359886.s001.docx]

# S1 Table: Full search strategy for a systematic review and meta-analysis for prevalence and genotype distribution of norovirus and rotavirus among children under five with acute diarrhea in lower-middle-income countries after rotavirus vaccine introduction from February 20 -28/2026

| No | Data Base | Keyword | **MeSH Term(Control vocabulary in PubMed)** |
| --- | --- | --- | --- |
| 1. | **PubMed** | Norovirus | ( "Norovirus/genetics"[Mesh] OR "Norovirus/isolation and purification"[Mesh] ) |
| 2. |  | Rotavirus | ( "Rotavirus/classification"[Mesh] OR "Rotavirus/genetics"[Mesh] OR "Rotavirus/isolation and purification"[Mesh] ) |
| 3. |  | Gastroenteritis | ( "Gastroenteritis/microbiology"[Mesh] OR "Gastroenteritis/virology"[Mesh] ) |
| 4. |  | Genotype | (((("Genotype"[Mesh]) OR "Molecular Epidemiology"[Mesh]) OR " Molecular Epidemiology "[Mesh]) OR "Epidemiology"[Mesh]) OR "Cross-Sectional Studies"[Mesh] |
| 5. |  | Diarrhea | ( "Diarrhea/classification"[Mesh] OR "Diarrhea/microbiology"[Mesh] OR "Diarrhea/prevention and control"[Mesh] OR "Diarrhea/virology"[Mesh] ) |
| 6. |  | Under Five children | child, preschool"[MeSH Terms] AND "Child"[MeSH Terms] |
|  |  | **#1 OR #2 AND #3 AND #4 AND #5 AND #6** | ((((( "Rotavirus/classification"[Mesh] OR "Rotavirus/genetics"[Mesh] OR "Rotavirus/isolation and purification"[Mesh] )) AND (( "Norovirus/genetics"[Mesh] OR "Norovirus/isolation and purification"[Mesh] ))) AND (( "Gastroenteritis"[Mesh] OR "Gastroenteritis/virology"[Mesh] ))) OR (( "Diarrhea/classification"[Mesh] OR "Diarrhea/microbiology"[Mesh] OR "Diarrhea/prevention and control"[Mesh] OR "Diarrhea/virology"[Mesh] ))) AND ("Child, Preschool"[Mesh] AND "Child"[Mesh]) |
|  | **Scopus** |  | TITLE-ABS-KEY ( "Norovirus" OR "Calicivirus" OR "Norwalk virus" OR "Rotavirus" OR "Reoviridae" ) AND TITLE-ABS-KEY ( Epidemiology OR Genotype OR Genogroups ) AND TITLE-ABS-KEY ( Gastroenteritis OR Diarrhea OR Prevalence ) AND TITLE-ABS-KEY ( Child OR Preschool ) |
|  | **Web of Science** |  | ((TS=("Rotavirus" OR "Rotavirus genetics" OR "Rotavirus isolation") AND TS=("Norovirus" OR "Norovirus genetics" OR "Norovirus isolation")) AND (TS="Gastroenteritis" OR TS="Gastroenteritis virology")) OR (TS=("Diarrhea" OR "Diarrhea classification" OR "Diarrhea microbiology" OR "Diarrhea prevention and control" OR "Diarrhea virology")) AND (TS="Child, Preschool" AND TS="Child") |
|  | **Embase** |  | ('norovirus'/exp OR 'calicivirus'/exp OR 'norwalk virus' OR norovirus OR calicivirus)  OR ('rotavirus'/exp OR 'reoviridae'/exp OR rotavirus OR reoviridae)  AND ('molecular epidemiology'/exp OR epidemiology OR genotype OR genogroup)  AND ('gastroenteritis'/exp OR diarrhea OR 'diarrhea'/exp)  AND ('child'/exp OR 'preschool child'/exp OR child OR 'preschool child') |
|  | **Science**  **direct** |  | (norovirus OR calicivirus OR "norwalk virus" OR rotavirus OR reoviridae) AND ("molecular epidemiology" OR epidemiology OR genotype OR genogroup) AND (gastroenteritis OR diarrhea) AND (child OR "preschool child") |
