# Supplementary material for: Prevalence and genotype distribution of norovirus and rotavirus among children under five with diarrheal infection in lower-middle-income countries after Rotavirus vaccine introduction: A systematic review and meta-analysis
Source: PLoS One. 2026 Oct 13;21(10):e0359886. doi: 10.1371/journal.pone.0359886 (PMC13660929; doi:10.1371/journal.pone.0359886)
Supplement: Supplementary file 3 [file pone.0359886.s002.docx]

S2 Table: List of LMICs with the year of rotavirus vaccine introduction for prevalence and genotype distribution of norovirus and rotavirus among children under five with acute diarrhea in lower-middle-income countries after rotavirus vaccine introduction, 2026.

| **No** | **Country** | **Year RV Started** |
| --- | --- | --- |
| 1 | Angola | 2014 |
| 2 | Bangladesh | 2018 |
| 3 | Benin | 2019 |
| 4 | Bhutan | 2019 |
| 5 | Bolivia | 2008 |
| 6 | Cambodia | 2025 |
| 7 | Cameroon | 2014 |
| 8 | Comoros | Not yet * |
| 9 | Congo, Rep. | 2014 |
| 10 | Côte d'Ivoire | 2017 |
| 11 | Djibouti | 2014 |
| 12 | Egypt | 2017 |
| 13 | Eswatini | 2015 |
| 14 | Ethiopia | 2013 |
| 15 | Ghana | 2012 |
| 16 | Guinea | Not yet * |
| 17 | Haiti | 2014 |
| 18 | Honduras | 2009 |
| 19 | India | 2016 |
| 20 | Jordan | 2015 |
| 21 | Kenya | 2014 |
| 22 | Kiribati | 2015 |
| 23 | Kyrgyz Republic | 2009 |
| 24 | Lao PDR | 2013 |
| 25 | Lebanon | 2018 |
| 26 | Lesotho | 2017 |
| 27 | Mauritania | 2014 |
| 28 | Micronesia, Fed. Sts. | 2009 |
| 29 | Morocco | 2010 |
| 30 | Myanmar | 2020 |
| 31 | Namibia | 2015 |
| 32 | Nepal | 2020 |
| 33 | Nicaragua | 2006 |
| 34 | Nigeria | 2022 |
| 35 | Pakistan | 2017 |
| 36 | Papua New Guinea | 2018 |
| 37 | Philippines | 2012 |
| 38 | São Tomé and Principe | 2016 |
| 39 | Senegal | 2014 |
| 40 | Solomon Islands | 2017 |
| 41 | Sri Lanka | 2020 |
| 42 | Tajikistan | 2015 |
| 43 | Tanzania | 2013 |
| 44 | Timor-Leste | 2020 |
| 45 | Tunisia | Not yet * |
| 46 | Uzbekistan | 2014 |
| 47 | Vanuatu | 2018 |
| 48 | Viet Nam | 2024 |
| 49 | West Bank and Gaza | 2016 |
| 50 | Zambia | 2013 |
| 51 | Zimbabwe | 2014 |
