# Supplementary material for: Prevalence and genotype distribution of norovirus and rotavirus among children under five with diarrheal infection in lower-middle-income countries after Rotavirus vaccine introduction: A systematic review and meta-analysis
Source: PLoS One. 2026 Oct 13;21(10):e0359886. doi: 10.1371/journal.pone.0359886 (PMC13660929; doi:10.1371/journal.pone.0359886)
Supplement: Supplementary file 5 [file pone.0359886.s004.docx]

S4 Table: Risk of bias and quality assessment results of the 45 studies included in the systematic review for prevalence and genotype distribution of norovirus and rotavirus among children under five with acute diarrhea in lower-middle-income countries after rotavirus vaccine introduction, 2026.

**A. Risk of bias and quality assessment Checklist for Prevalence Studies**

| **Code** | **Items** | **Response categories** | | | |
| --- | --- | --- | --- | --- | --- |
|  |  | **Yes (Y)** | **No (N)** | **Unclear (U)** | **Not applicable (NA)** |
| QA 1 | Was the sample frame appropriate to address the target population? |  |  |  |  |
| QA 2 | Were study participants sampled in an appropriate way? |  |  |  |  |
| QA 3 | Was the sample size adequate? |  |  |  |  |
| QA 4 | Were the study subjects and the setting described in detail? |  |  |  |  |
| QA 5 | Was the data analysis conducted with sufficient coverage of the identified sample? |  |  |  |  |
| QA 6 | Were valid methods used for the identification of the condition? |  |  |  |  |
| QA 7 | Was the condition measured in a standard, reliable way for all participants? |  |  |  |  |
| QA 8 | Was there appropriate statistical analysis? |  |  |  |  |
|  | Was the response rate adequate, and if not, was the low response rate managed appropriately? |  |  |  |  |

**B. Quality check for identified studies**

| 1. | Author, year | Country | Study design | Q1 | Q2 | Q3 | Q4 | Q5 | Q6 | Q7 | Q8 | Q9 | % Yes | Quality | Decision |
| --- | --- | --- | --- | --- | --- | --- | --- | --- | --- | --- | --- | --- | --- | --- | --- |
| 2. | Abubakar et al,2023 | Nigeria | Cross-sectional | **Y** | **Y** | **Y** | **Y** | **Y** | **Y** | **Y** | **Y** | **Y** | 100 | High | Include |
| 3. | Alam et al, 2015 | Pakistan | Cross-sectional | **Y** | **Y** | **Y** | **Y** | **Y** | **Y** | **Y** | **Y** | **Y** | 100 | High | Include |
| 4. | Arowolo, K.O, et al, 2023 | Nigeria | cross-sectional | **Y** | **Y** | **Y** | **Y** | **Y** | **Y** | **Y** | **Y** | **Y** | 100 | High | Include |
| 5. | S.K. Dey et al, 2007 | Bangladesh | cross-sectional | **Y** | **Y** | **Y** | **Y** | **Y** | **Y** | **Y** | **Y** | **Y** | 100 | High | Include |
| 6. | El Qazoui et a, 2024 | Morocco | cross-sectional | **Y** | **Y** | **Y** | **Y** | **Y** | **Y** | **Y** | **Y** | **Y** | 100 | High | Include |
| 7. | El Qazoui et a, 2024 | Morocco | cross-sectional | **Y** | **Y** | **Y** | **Y** | **Y** | **Y** | **Y** | **Y** | **Y** | 100 | High | Include |
| 8. | A. Esteves et al, 2018 | Angola | cross-sectional | **Y** | **Y** | **Y** | **Y** | **Y** | **Y** | **Y** | **Y** | **Y** | 100 | High | Include |
| 9. | A. Gelaw et al, 2019 | Ethiopia | cross-sectional | **Y** | **Y** | **Y** | **N** | **Y** | **Y** | **Y** | **Y** | **Y** | 88.8 | High | Include |
| 10. | T.N. Hoa-Tran et al,2015 | Nepal | cross-sectional | **Y** | **Y** | **Y** | **Y** | **Y** | **Y** | **Y** | **Y** | **Y** | 100 | High | Include |
| 11. | Gopalkrishna et al,2021 | India | cross-sectional | **Y** | **N** | **Y** | **N** | **Y** | **Y** | **Y** | **Y** | **Y** | 77.7 | Moderate | Include |
| 12. | Gopalkrishna et al,2021 | India | cross-sectional | **Y** | **N** | **Y** | **N** | **Y** | **Y** | **Y** | **Y** | **Y** | 77.7 | Moderate | Include |
| 13. | Japhet et al, 2012 | Nigeria | cross-sectional | **Y** | **N** | **Y** | **Y** | **Y** | **Y** | **Y** | **Y** | **Y** | 77.7 | Moderate | Include |
| 14. | Kebe et al, 2021 | Senegal | cross-sectional | **Y** | **N** | **Y** | **Y** | **Y** | **Y** | **Y** | **Y** | **Y** | 77.7 | Moderate | Include |
| 15. | R. Kulkarni et al, 2016 | India | cross-sectional | **Y** | **Y** | **Y** | **Y** | **Y** | **Y** | **Y** | **Y** | **Y** | 100 | High | Include |
| 16. | S.E. Lekana-Douki et al, 2015 | Gabon | cross-sectional | **Y** | **Y** | **Y** | **Y** | **Y** | **N** | **N** | **Y** | **Y** | 66.6 | Moderate | Include |
| 17. | M. Lo et al,2021 | India | cross-sectional | **Y** | **Y** | **Y** | **N** | **Y** | **Y** | **Y** | **Y** | **Y** | 88.8 | High | Include |
| 18. | Melhem NM et al., 2016 | Lebanon | cross-sectional | **Y** | **Y** | **Y** | **N** | **Y** | **Y** | **Y** | **Y** | **Y** | 88.8 | High | Include |
| 19. | Menon et al, 2013(short report ) | India | cross-sectional | **Y** | **N** | **Y** | **N** | **Y** | **N** | **N** | **Y** | **Y** | 55.5 | Moderate | Include |
| 20. | Mugyia et al., 2019 | Cameroon | cross-sectional | **Y** | **Y** | **Y** | **Y** | **Y** | **Y** | **Y** | **Y** | **Y** | 100 | High | Include |
| 21. | Ortiz-Quintero et al ,2024 | Honduras | Cross sectional | **Y** | **Y** | **Y** | **Y** | **Y** | **Y** | **Y** | **Y** | **Y** | 100 | High | Include |
| 22. | Satter et al 2022 | Bangladesh | Cross sectional | **Y** | **Y** | **Y** | **N** | **Y** | **Y** | **Y** | **Y** | **N** | 77.7 | Moderate | Include |
| 23. | N. Sharif et al, 2023 | Bangladesh | Cross sectional | **Y** | **Y** | **Y** | **Y** | **Y** | **Y** | **Y** | **Y** | **Y** | 100 | High | Include |
| 24. | Wangchu et al, 2017 | Bhutan | Cross sectional | **Y** | **Y** | **Y** | **Y** | **Y** | **Y** | **Y** | **Y** | **Y** | 100 | High | Include |
| 25. | A. Abebe et a, 2018 | Ethiopia | Cross sectional | Y | Y | Y | Y | Y | Y | Y | Y | Y | 100 | High | Include |
| 26. | Kumar et al, 2022 | India | Cross sectional | Y | Y | Y | Y | Y | Y | Y | Y | Y | 100 | High | Include |
| 27. | Namboodiripad1, 2025 | India | Cross sectional | Y | Y | Y | Y | Y | Y | Y | Y | Y | 100 | High | Include |
| 28. | Kumar, et al, 2025 | India | Cross sectional | Y | Y | Y | N | Y | Y | Y | Y | Y | 88.8 | High | Include |
| 29. | Nyamanga et al, 2024 | Kenya | Cross sectional | Y | Y | Y | Y | Y | Y | Y | Y | Y | 100 | High | Include |
| 30. | Singh et al, 2025 | India | Cross sectional | Y | Y | Y | N | Y | Y | Y | Y | Y | 88.8 | High | Include |
| 31. | Gurung, et al, | India | Cross sectional | Y | Y | Y | N | Y | Y | Y | Y | Y | 88.8 | High | Include |
| 32. | Jafet | Honduras | Cross sectional | Y | Y | Y | N | Y | Y | Y | Y | Y | 88.8 | High | Include |
| 33. | Jain, et al, 2021 | India | Cross sectional | Y | Y | Y | Y | Y | Y | Y | Y | Y | 100 | High | Include |
| 34. | SimwakaI eat al , 2021 | Zambia | Cross sectional | Y | Y | Y | Y | Y | Y | Y | Y | Y | 100 | High | Include |
| 35. | Engade et al, 2025 | India | Cross sectional | Y | Y | Y | N | Y | Y | Y | Y | Y | 88.8 | High | Include |
| 36. | Mohanty et al, 2025 | India | Cross sectional | Y | Y | Y | N | Y | Y | Y | Y | N | 77.7 | Moderate | Include |
| 37. | Mohanty et alb, 2025 | India | Cross sectional | Y | Y | Y | N | Y | Y | Y | Y | N | 77.7 | Moderate | Include |
| 38. | Rajani et al, 2025 | India | cross-sectional | Y | Y | Y | N | Y | Y | Y | Y | N | 77.7 | Moderate | Include |
| 39. | Rawal et al, 2021 | India | surveillance | Y | Y | Y | N | Y | Y | Y | Y | N | 77.7 | Moderate | Include |
| 40. | Pradhan, et al, 2021 | India | prospective | Y | Y | Y | Y | Y | Y | Y | Y | N | 88.8 | High | Include |
| 41. | Vyas et al, 2025 | India | prospective, hospital-based surveillance | Y | Y | Y | Y | Y | Y | Y | Y | N | 88.8 | High | Include |
| 42. | India et al 2025 | India | observational study | Y | Y | Y | Y | Y | Y | Y | Y | Y | 88.8 | High | Include |
| 43. | Redda et al. 2025 | Ethiopia | Cross sectional | Y | Y | Y | Y | Y | Y | Y | Y | Y | 100 | High | Include |
| 44. | Rahman et at.,2016 | Bangladesh | surveillance |  |  |  |  |  |  |  |  | Y | 77.7 | Moderate | Include |
| 45. | Tegegne D, et al,2024 | Ethiopia | Cross sectional | Y | Y | Y |  | Y | Y | Y | Y | Y | 77.7 | Moderate | Include |
