# Supplementary material for: Prevalence and genotype distribution of norovirus and rotavirus among children under five with diarrheal infection in lower-middle-income countries after Rotavirus vaccine introduction: A systematic review and meta-analysis
Source: PLoS One. 2026 Oct 13;21(10):e0359886. doi: 10.1371/journal.pone.0359886 (PMC13660929; doi:10.1371/journal.pone.0359886)
Supplement: Supplementary file 6 [file pone.0359886.s006.docx]

**Response to Academic Editor**

**Journal Requirements:**

When submitting your revision, we need you to address these additional requirements.

1. Please ensure that your manuscript meets PLOS ONE's style requirements, including those for file naming. The PLOS ONE style templates can be found at

[https://journals.plos.org/plosone/s/file?id=wjVg/PLOSOne_formatting_sample_main_body.pdf](https://track.editorialmanager.com/CL0/https:%2F%2Fjournals.plos.org%2Fplosone%2Fs%2Ffile%3Fid=wjVg%2FPLOSOne_formatting_sample_main_body.pdf/1/010f01a017f5c09e-88130aca-88c6-4446-abd8-0149916b16ea-000000/ikTWHYKGVErebMEUdEUsFsEMw5LSV49WWQaeeLAjK84=258) and

[https://journals.plos.org/plosone/s/file?id=ba62/PLOSOne_formatting_sample_title_authors_affiliations.pdf](https://track.editorialmanager.com/CL0/https:%2F%2Fjournals.plos.org%2Fplosone%2Fs%2Ffile%3Fid=ba62%2FPLOSOne_formatting_sample_title_authors_affiliations.pdf/1/010f01a017f5c09e-88130aca-88c6-4446-abd8-0149916b16ea-000000/MK7mZjZ_U9y1AgXEyLepPJoATw9OnCbRS8tcSL1E3uw=258)

**Response:** We sincerely thank the Academic Editor for your time, effort, and valuable guidance. We have carefully revised the manuscript in accordance with the journal’s requirements and have addressed the necessary corrections. We kindly request that you consider the revised manuscript for further evaluation.

2. In the online submission form you indicate that your data is not available for proprietary reasons and have provided a contact point for accessing this data. Please note that your current contact point is a co-author on this manuscript. According to our Data Policy, the contact point must not be an author on the manuscript and must be an institutional contact, ideally not an individual. Please revise your data statement to a non-author institutional point of contact, such as a data access or ethics committee, and send this to us via return email. Please also include contact information for the third-party organization, and please include the full citation of where the data can be found.

**Response:** We thank academic editor. We have revised the Data Availability Statement in accordance with the PLOS ONE Data Availability Policy. Since this study is a systematic review and meta-analysis based on data extracted from previously published studies, we have clarified the source and availability of the data and removed the author as the data-access contact.

3. Please ensure that you refer to Figures 9 and 12 to 16 in your text as, if accepted, production will need this reference to link the reader to the figure.

**Response:** Thank you for pointing this out. We have revised the manuscript to ensure that Figures 9 and 12–16 are appropriately referred to in the text.

4. Please include captions for your Supporting Information files at the end of your manuscript, and update any in-text citations to match accordingly. Please see our Supporting Information guidelines for more information: [http://journals.plos.org/plosone/s/supporting-information](http://track.editorialmanager.com/CL0/http:%2F%2Fjournals.plos.org%2Fplosone%2Fs%2Fsupporting-information/1/010f01a017f5c09e-88130aca-88c6-4446-abd8-0149916b16ea-000000/OMIxWOCyghRlwfLodBI_GQBhKqzegy4RkS-VhnbqQak=258).

**Response:** we have included all the caption at the end of the manuscript.

5. If the reviewer comments include a recommendation to cite specific previously published works, please review and evaluate these publications to determine whether they are relevant and should be cited. There is no requirement to cite these works unless the editor has indicated otherwise.

**Response:** Okay thank you.

**Response Reviewer #1**

1. Overall, this manuscript addresses a highly relevant public health topic, particularly given the increasing importance of norovirus following the introduction of rotavirus vaccination. The research question is pertinent, and the study employs an appropriate systematic review and meta-analysis methodology. However, several methodological, statistical, and writing issues reduce the overall strength of the conclusions.

- **Response:** Thank you so much reviewer for the constructive evaluation of our manuscript. We appreciate the positive assessment of the relevance of the research question and the appropriateness of this manuscript. We have carefully considered all comments and have revised the manuscript accordingly. Please look at the newly revised manuscript.

2. The manuscript would benefit from careful language editing by a native English speaker or a professional scientific editing service. There are numerous grammatical errors and awkward sentence constructions throughout the text. For example, the sentence “Approximately one in five children presenting with diarrheal illness infected...” is grammatically incorrect and should be revised. In addition, several expressions, particularly “This systematic review,” are repeated excessively, making the text unnecessarily repetitive.

- **Response:** We thank the reviewer for this important observation. We agree that the previous version contained grammatical errors, awkward sentence constructions, and some repetitive expressions. We have therefore carefully revised the manuscript to improve grammar, readability, clarity, and scientific style. Please look at the newly uploaded manuscript.

3. Some methodological aspects also require clarification. The authors do not describe how studies reporting zero prevalence were handled in the meta-analysis, which is an important issue when pooling prevalence estimates.

- **Response:** We thank the reviewer for highlighting this important methodological issue. We agree that the treatment of studies reporting zero prevalence should be explicitly described in a prevalence meta-analysis. We have clarified the statistical methods used to handle zero-prevalence studies in the revised manuscript.

4. Restricting the search to English-language publications may have introduced language bias and should be acknowledged more explicitly.

- **Response:** We agree with the reviewer. Restricting the search to English-language publications may have resulted in the exclusion of potentially relevant studies published in other languages and may therefore have introduced language bias. We have mentioned it in the limitation part please look at the newly uploaded manuscript.

5. Furthermore, combining studies that used different diagnostic methods, such as RT-PCR and ELISA, is likely to have contributed to the substantial heterogeneity observed. A meta-regression including diagnostic method as a covariate would help determine whether the laboratory technique explains part of the between-study variability.

- **Response:** We agree that differences in diagnostic methods, including RT-PCR and ELISA, may contribute to between-study heterogeneity because these methods can differ in diagnostic performance and analytical characteristics. We therefore re-evaluated the diagnostic methods used across the included studies and considered diagnostic method as a potential source of heterogeneity. Where the number of studies and the distribution of diagnostic methods were sufficient to support a meaningful analysis, we performed meta-regression with diagnostic method as a covariate.

**Response Reviewer #2**

We sincerely thank Reviewer 2 for the time, constructive comments, and valuable suggestions. Your feedback has helped us improve the quality and clarity of the manuscript. We have carefully addressed all of your comments and suggestions and revised the manuscript accordingly. We also appreciate your efforts in highlighting both the strengths and weaknesses of our manuscript. Please kindly review the newly uploaded revised manuscript.

## 1. **General Introduction & Summary**

**Weaknesses.** Several numerical inconsistencies recur across sections and should be reconciled: the overall I² is variously reported as 98.85%, 98.72%, and 98.67%; GII pooled prevalence appears as both 76% and 75%; the GI prevalence (23%) is paired with a CI (10–17%) that does not contain the point estimate (Figure 14 gives 10–37%); and the stated rotavirus prevalence range (“17.90–24.10%”) does not match several Table 1 entries as high as 46.50%. Figure citations in the sensitivity-analysis subsection also appear mismatched against the actual figure captions, and a citation string in the genotype section is garbled, likely from a page-break artifact.

Beyond these, a more substantive point: given I² near 98–99% throughout, a single pooled headline figure (18%) may understate how disparate the underlying data are, even with subgroup analysis performed. Notably, the manuscript’s claim that “one study reported

both viruses” appears incorrect Table 1 shows two (El Qazoui et al., 2024 and Gopalkrishna et al., 2021, each listed under separate norovirus/rotavirus rows). We would also flag that nearly half of included studies (20/45) originate from India, which limits generalizability to other LMIC regions; that the English-only search restriction may have compounded the detected publication bias by excluding French/Spanish/Portuguese-language literature; and that pooling cross-sectional and surveillance designs together, despite differing methodologies, risks obscuring real differences in burden. Finally, if the Data Availability Statement restricts data to “available upon request,” this would not meet PLOS ONE’s minimal-dataset policy we could not verify this wording against the material provided and would ask the editor to confirm with the authors.

None of these issues undermine the manuscript’s core validity or contribution; they are correctable through text and data reconciliation, consistent with Accept with Text Revisions. However, several of the items above are substantive data-accuracy checks, not merely stylistic ones, and should be resolved before production.

## 2. **Line-by-Line Revisions & Language Corrections**

*Representative* ***examples*** *are given for each manuscript section; this is not an exhaustive list. Citation-bracket spacing (e.g., “word[12]” instead of “word [12]”) recurs dozens of times throughout and should be corrected globally.*

### Abstract

| Line | Original Text | Suggested Revision | Justification | **Response** |
| --- | --- | --- | --- | --- |
| 42 | “children presenting with diarrheal illness infected with at least one of these viruses” | “…illness were infected with at least one of these viruses” | Missing auxiliary verb. | **We have corrected it. kindly review the newly uploaded revised manuscript.** |
| 45–  46 | “monitor genotype shifts among population” | “monitor genotype shifts within the population” | Missing article/wrong  preposition. | **Corrected** |

### **Introduction**

| **Line** | **Original Text** | **Suggested Revision** | **Justification** | **Response** |
| --- | --- | --- | --- | --- |
| 64 | “Norovirus (NoV) is belongs to the Caliciviridae  family…single stranded, no enveloped” | “Norovirus (NoV) belongs to the Caliciviridae  family…single-stranded,  non-enveloped” | Double verb; incorrect terminology/hyphenation. | **Corrected** |
| 78 | “especially on under five children’s” | “especially on children under five” | Misplaced apostrophe, wrong word order. | **Corrected** |
| Line | Original Text | Suggested Revision | Justification | **Corrected** |
| 87 | “to the best of my knowledge” | “to the best of our knowledge” | Multi-author manuscript  should use “our.” | **Corrected** |

### **Methods**

| **Line** | **Original Text** | **Suggested Revision** | **Justification** | **Response** |
| --- | --- | --- | --- | --- |
| 126–  127 | “the search result were imported into Rayyan(AI-Powered…” | “the search results were imported into Rayyan (AI-Powered…” | Subject–verb agreement; missing space. | **Corrected** |
| 140–  141 | “The overall quality of each eligible article were  evaluated” | “…was evaluated” | Subject–verb agreement. | **Corrected** |

### **Results**

| **Line** | **Original Text** | **Suggested Revision** | **Justification** | **Response** |
| --- | --- | --- | --- | --- |
| 164–  165 | “Finally,45 articles were included…” | “Finally, 45 articles were included…” | Missing space after comma. | **Corrected** |
| 190–  191 | “…one study reported both viruses” | “…two studies reported both viruses” | Factual error Table 1 shows El Qazoui et al. 2024 and Gopalkrishna et  al. 2021 each reporting both viruses. |  |
| 266–  267 | GI prevalence  “23% (95% CI: 10–  17%)” | “23% (95% CI:  10–37%)” *(verify)* | CI does not contain the point estimate; inconsistent with Figure 14. | **Corrected** |

### **Discussion**

| **Line** | **Original Text** | **Suggested Revision** | **Justification** | **Response** |
| --- | --- | --- | --- | --- |
| 278–  280 | “Transmission often a rises  periodically…how the infection spread in these counties” | “…often arises  periodically…how infection spreads in these countries” | Typos; subject–verb agreement. | **Corrected** |
| 320–  322 | “…84.6% [81], his genotype is considered…” | “…84.6% [81]. This  genotype is considered…” | Typo (“his” for “This”); comma  splice. | **Corrected** |

### **Conclusions, Recommendations & References**

| **Line** | **Original Text** | **Suggested Revision** | **Justification** | **Response** |
| --- | --- | --- | --- | --- |
| 356–  357 | “This This systematic review and meta-analysis synthesize evidence…” | “This systematic review and meta-analysis  synthesizes evidence…” | Duplicated word; subject–verb agreement. | **Corrected** |
| Ref 64 | “Children s Hospital in 2016, in Lusaka Zambia” | “Children’s Hospital in 2016, in Lusaka, Zambia” | Missing apostrophe and comma. | **Corrected** |
| Table 1,  Row 34 | “SimwakaI eat al , 2021” | “Simwaka et al., 2021” | Typo in author name and “et al.” | **Corrected** |

**Thank all for your constructive and helpful comments.**
