# Supplementary material for: Prevalence and genotype distribution of norovirus and rotavirus among children under five with diarrheal infection in lower-middle-income countries after Rotavirus vaccine introduction: A systematic review and meta-analysis
Source: PLoS One. 2026 Oct 13;21(10):e0359886. doi: 10.1371/journal.pone.0359886 (PMC13660929; doi:10.1371/journal.pone.0359886)
Supplement: Supplementary file 7 [file pone.0359886.s007.docx]

**Response to Reviewers**

We sincerely thank the reviewers and the Academic Editor for their careful evaluation of our revised manuscript and for their constructive and valuable comments. We greatly appreciate Reviewer 1’s recommendation for acceptance and Reviewer 2’s positive assessment of the revised manuscript. We have carefully considered the remaining minor issues raised by Reviewer 2 and have addressed each point as detailed below.

**Response to Reviewer 2**

**Comment 1:**
“Please submit a clean file free of tracked-changes artifacts.”

**Response:** Thank you for pointing this out. We have carefully reviewed the revised manuscript and prepared a clean version free of tracked changes, comments, and other revision artifacts. The clean manuscript has been uploaded as requested.

**Comment 2:**
“Reconcile the I² value throughout.”

**Response:** Thank you for raising this point. We carefully checked all reported I² values throughout the manuscript. We confirmed that the differences in I² values do not represent inconsistencies or errors; rather, they reflect the different levels of heterogeneity observed across the overall meta-analysis and the various subgroup analyses. Because each subgroup analysis is based on a different set of studies, the corresponding I² values naturally differ. We have nevertheless carefully reviewed the presentation of all I² values throughout the manuscript and ensured that each value corresponds correctly to its respective overall or subgroup analysis and is reported consistently with the statistical results.

**Comment 3:**
“Either add the promised diagnostic-method meta-regression or remove that from the response letter.”

**Response:** We sincerely thank the reviewer for this important clarification and apologize for any confusion caused by our previous response. Following the reviewer’s suggestion, we have removed the statement regarding the promised diagnostic-method meta-regression from the response letter. We have also carefully reviewed the manuscript and confirmed that it contains no unsupported claims regarding this analysis. We greatly appreciate the reviewer’s careful attention to this matter, which has helped us improve the clarity and accuracy of our response.

**Comment 4:**
“Proofread the specific lines noted, several of which were marked ‘Corrected’ but remain unchanged.”

**Response:** We sincerely appreciate the reviewer for identifying this issue. We have rechecked all the specific lines and comments raised in the previous review round, particularly those for which we had indicated “Corrected.” We carefully compared the reviewer’s comments, our previous responses, and the revised manuscript, and have now made the necessary corrections wherever they were still missing. The manuscript has also been thoroughly proofread to ensure that all stated corrections are accurately reflected in the final version.
