# Supplementary material for: Prevalence and genotype distribution of norovirus and rotavirus among children under five with diarrheal infection in lower-middle-income countries after Rotavirus vaccine introduction: A systematic review and meta-analysis
Source: PLoS One. 2026 Oct 13;21(10):e0359886. doi: 10.1371/journal.pone.0359886 (PMC13660929; doi:10.1371/journal.pone.0359886)
Supplement: Peer review history

# Peer review: Decision Letter 0

**DOI:** 10.1371/journal.pone.0359886.r001

**Authors:** Ricardo Q. Gurgel

18 Aug 2026

Dear Dr. Ayele,

Please submit your revised manuscript by Oct 17 2026 11:59PM. If you will need more time than this to complete your revisions, please reply to this message or contact the journal office at plosone@plos.org. When you're ready to submit your revision, log on to https://www.editorialmanager.com/pone/ and select the 'Submissions Needing Revision' folder to locate your manuscript file.

- A letter that responds to each point raised by the academic editor and reviewer(s). You should upload this letter as a separate file labeled 'Response to Reviewers'.
- A marked-up copy of your manuscript that highlights changes made to the original version. You should upload this as a separate file labeled 'Revised Manuscript with Track Changes'.
- An unmarked version of your revised paper without tracked changes. You should upload this as a separate file labeled 'Manuscript'.

If you would like to make changes to your financial disclosure, please include your updated statement in your cover letter. Guidelines for resubmitting your figure files are available below the reviewer comments at the end of this letter.

If applicable, we recommend that you deposit your laboratory protocols in protocols.io to enhance the reproducibility of your results. Protocols.io assigns your protocol its own identifier (DOI) so that it can be cited independently in the future. For instructions see: https://journals.plos.org/plosone/s/submission-guidelines#loc-laboratory-protocols. Additionally, PLOS One offers an option for publishing peer-reviewed Lab Protocol articles, which describe protocols hosted on protocols.io. Read more information on sharing protocols at https://plos.org/protocols?utm_medium=editorial-email&utm_source=authorletters&utm_campaign=protocols.

As the corresponding author, your ORCID iD is verified in the submission system and will appear in the published article. PLOS supports the use of ORCID, and we encourage all coauthors to register for an ORCID iD and use it as well. Please encourage your coauthors to verify their ORCID iD within the submission system before final acceptance, as unverified ORCID iDs will not appear in the published article. *Only*  the individual author can complete the verification step; PLOS staff *cannot*  verify ORCID iDs on behalf of authors.

We look forward to receiving your revised manuscript.

Kind regards,

Ricardo Q. Gurgel, PhD

Academic Editor

PLOS One

**Journal Requirements:**

When submitting your revision, we need you to address these additional requirements.

1. Please ensure that your manuscript meets PLOS ONE's style requirements, including those for file naming. The PLOS ONE style templates can be found at

https://journals.plos.org/plosone/s/file?id=wjVg/PLOSOne_formatting_sample_main_body.pdf and

https://journals.plos.org/plosone/s/file?id=ba62/PLOSOne_formatting_sample_title_authors_affiliations.pdf

2. In the online submission form you indicate that your data is not available for proprietary reasons and have provided a contact point for accessing this data. Please note that your current contact point is a co-author on this manuscript. According to our Data Policy, the contact point must not be an author on the manuscript and must be an institutional contact, ideally not an individual. Please revise your data statement to a non-author institutional point of contact, such as a data access or ethics committee, and send this to us via return email. Please also include contact information for the third party organization, and please include the full citation of where the data can be found.

3. Please ensure that you refer to Figures 9 and  12 to 16 in your text as, if accepted, production will need this reference to link the reader to the figure.

4. Please include captions for your Supporting Information files at the end of your manuscript, and update any in-text citations to match accordingly. Please see our Supporting Information guidelines for more information: http://journals.plos.org/plosone/s/supporting-information.

5. If the reviewer comments include a recommendation to cite specific previously published works, please review and evaluate these publications to determine whether they are relevant and should be cited. There is no requirement to cite these works unless the editor has indicated otherwise.

[Note: HTML markup is below. Please do not edit.]

Reviewers' comments:

Reviewer's Responses to Questions

**Comments to the Author**

1. Is the manuscript technically sound, and do the data support the conclusions?

Reviewer #1: Partly

Reviewer #2: Yes

**********

2. Has the statistical analysis been performed appropriately and rigorously?

Reviewer #1: Yes

Reviewer #2: Yes

**********

3. Have the authors made all data underlying the findings in their manuscript fully available?

The PLOS Data policy

Reviewer #1: Yes

Reviewer #2: Yes

**********

4. Is the manuscript presented in an intelligible fashion and written in standard English?

Reviewer #1: No

Reviewer #2: Yes

**********

Reviewer #1: Overall, this manuscript addresses a highly relevant public health topic, particularly given the increasing importance of norovirus following the introduction of rotavirus vaccination. The research question is pertinent, and the study employs an appropriate systematic review and meta-analysis methodology. However, several methodological, statistical, and writing issues reduce the overall strength of the conclusions.

The manuscript would benefit from careful language editing by a native English speaker or a professional scientific editing service. There are numerous grammatical errors and awkward sentence constructions throughout the text. For example, the sentence "Approximately one in five children presenting with diarrheal illness infected..." is grammatically incorrect and should be revised. In addition, several expressions, particularly "This systematic review", are repeated excessively, making the text unnecessarily repetitive.

Some methodological aspects also require clarification. The authors do not describe how studies reporting zero prevalence were handled in the meta-analysis, which is an important issue when pooling prevalence estimates. Restricting the search to English-language publications may have introduced language bias and should be acknowledged more explicitly. Furthermore, combining studies that used different diagnostic methods, such as RT-PCR and ELISA, is likely to have contributed to the substantial heterogeneity observed. A meta-regression including diagnostic method as a covariate would help determine whether the laboratory technique explains part of the between-study variability.

Reviewer #2: Attached is the full report,

Herein, the authors present a systematic review and meta-analysis of the pooled prevalence and genotype distribution of norovirus and rotavirus among children under five with diarrheal infection across 45 studies (35,704 children) from low- and middle-income countries (LMICs), restricted to the period following national rotavirus vaccine introduction. Using a random-effects (REML) model, the overall pooled prevalence was 18% (95% CI: 15–21%), with rotavirus (24%) more prevalent than norovirus (13%). Heterogeneity was substantial throughout (I² ≈ 98–99%) and was explored via subgroup and meta-regression analyses. GII, particularly GII.4, was the dominant norovirus genotype, while G3P[8] and G1[P8] led among rotavirus genotypes. The authors conclude that both viruses remain a significant burden in LMICs post-vaccination and call for continued genomic surveillance.

**********

what does this mean?). If published, this will include your full peer review and any attached files.

If you choose “no”, your identity will remain anonymous but your review may still be made public.

**Do you want your identity to be public for this peer review?**  For information about this choice, including consent withdrawal, please see our Privacy Policy

Reviewer #1: **Yes:**  CATHARINA CORREA COSTA

Reviewer #2: No

**********

[NOTE: If reviewer comments were submitted as an attachment file, they will be attached to this email and accessible via the submission site. Please log into your account, locate the manuscript record, and check for the action link "View Attachments". If this link does not appear, there are no attachment files.]

To ensure your figures meet our technical requirements, please review our figure guidelines: https://journals.plos.org/plosone/s/figures

You may also use PLOS’s free figure tool, NAAS, to help you prepare publication quality figures: https://journals.plos.org/plosone/s/figures#loc-tools-for-figure-preparation.

NAAS will assess whether your figures meet our technical requirements by comparing each figure against our figure specifications.

Attachment Submitted filename: Peer_Review_Norovirus_Rotavirus_Manuscript.pdf

# Author comment: Author response to Decision Letter 1

**DOI:** 10.1371/journal.pone.0359886.r002

1 Sep 2026

Response to Academic Editor

Journal Requirements:

When submitting your revision, we need you to address these additional requirements.

1. Please ensure that your manuscript meets PLOS ONE's style requirements, including those for file naming. The PLOS ONE style templates can be found at

https://journals.plos.org/plosone/s/file?id=wjVg/PLOSOne_formatting_sample_main_body.pdf and

https://journals.plos.org/plosone/s/file?id=ba62/PLOSOne_formatting_sample_title_authors_affiliations.pdf

Response: We sincerely thank the Academic Editor for your time, effort, and valuable guidance. We have carefully revised the manuscript in accordance with the journal’s requirements and have addressed the necessary corrections. We kindly request that you consider the revised manuscript for further evaluation.

2. In the online submission form you indicate that your data is not available for proprietary reasons and have provided a contact point for accessing this data. Please note that your current contact point is a co-author on this manuscript. According to our Data Policy, the contact point must not be an author on the manuscript and must be an institutional contact, ideally not an individual. Please revise your data statement to a non-author institutional point of contact, such as a data access or ethics committee, and send this to us via return email. Please also include contact information for the third-party organization, and please include the full citation of where the data can be found.

Response: We thank academic editor. We have revised the Data Availability Statement in accordance with the PLOS ONE Data Availability Policy. Since this study is a systematic review and meta-analysis based on data extracted from previously published studies, we have clarified the source and availability of the data and removed the author as the data-access contact.

3. Please ensure that you refer to Figures 9 and 12 to 16 in your text as, if accepted, production will need this reference to link the reader to the figure.

Response: Thank you for pointing this out. We have revised the manuscript to ensure that Figures 9 and 12–16 are appropriately referred to in the text.

4. Please include captions for your Supporting Information files at the end of your manuscript, and update any in-text citations to match accordingly. Please see our Supporting Information guidelines for more information: http://journals.plos.org/plosone/s/supporting-information.

Response: we have included all the caption at the end of the manuscript.

5. If the reviewer comments include a recommendation to cite specific previously published works, please review and evaluate these publications to determine whether they are relevant and should be cited. There is no requirement to cite these works unless the editor has indicated otherwise.

Response: Okay thank you.

Response Reviewer #1

1. Overall, this manuscript addresses a highly relevant public health topic, particularly given the increasing importance of norovirus following the introduction of rotavirus vaccination. The research question is pertinent, and the study employs an appropriate systematic review and meta-analysis methodology. However, several methodological, statistical, and writing issues reduce the overall strength of the conclusions.

� Response: Thank you so much reviewer for the constructive evaluation of our manuscript. We appreciate the positive assessment of the relevance of the research question and the appropriateness of this manuscript. We have carefully considered all comments and have revised the manuscript accordingly. Please look at the newly revised manuscript.

2. The manuscript would benefit from careful language editing by a native English speaker or a professional scientific editing service. There are numerous grammatical errors and awkward sentence constructions throughout the text. For example, the sentence “Approximately one in five children presenting with diarrheal illness infected...” is grammatically incorrect and should be revised. In addition, several expressions, particularly “This systematic review,” are repeated excessively, making the text unnecessarily repetitive.

� Response: We thank the reviewer for this important observation. We agree that the previous version contained grammatical errors, awkward sentence constructions, and some repetitive expressions. We have therefore carefully revised the manuscript to improve grammar, readability, clarity, and scientific style. Please look at the newly uploaded manuscript.

3. Some methodological aspects also require clarification. The authors do not describe how studies reporting zero prevalence were handled in the meta-analysis, which is an important issue when pooling prevalence estimates.

� Response: We thank the reviewer for highlighting this important methodological issue. We agree that the treatment of studies reporting zero prevalence should be explicitly described in a prevalence meta-analysis. We have clarified the statistical methods used to handle zero-prevalence studies in the revised manuscript.

4. Restricting the search to English-language publications may have introduced language bias and should be acknowledged more explicitly.

� Response: We agree with the reviewer. Restricting the search to English-language publications may have resulted in the exclusion of potentially relevant studies published in other languages and may therefore have introduced language bias. We have mentioned it in the limitation part please look at the newly uploaded manuscript.

5. Furthermore, combining studies that used different diagnostic methods, such as RT-PCR and ELISA, is likely to have contributed to the substantial heterogeneity observed. A meta-regression including diagnostic method as a covariate would help determine whether the laboratory technique explains part of the between-study variability.

� Response: We agree that differences in diagnostic methods, including RT-PCR and ELISA, may contribute to between-study heterogeneity because these methods can differ in diagnostic performance and analytical characteristics. We therefore re-evaluated the diagnostic methods used across the included studies and considered diagnostic method as a potential source of heterogeneity. Where the number of studies and the distribution of diagnostic methods were sufficient to support a meaningful analysis, we performed meta-regression with diagnostic method as a covariate.

Response Reviewer #2

We sincerely thank Reviewer 2 for the time, constructive comments, and valuable suggestions. Your feedback has helped us improve the quality and clarity of the manuscript. We have carefully addressed all of your comments and suggestions and revised the manuscript accordingly. We also appreciate your efforts in highlighting both the strengths and weaknesses of our manuscript. Please kindly review the newly uploaded revised manuscript.

1. General Introduction & Summary

Weaknesses. Several numerical inconsistencies recur across sections and should be reconciled: the overall I² is variously reported as 98.85%, 98.72%, and 98.67%; GII pooled prevalence appears as both 76% and 75%; the GI prevalence (23%) is paired with a CI (10–17%) that does not contain the point estimate (Figure 14 gives 10–37%); and the stated rotavirus prevalence range (“17.90–24.10%”) does not match several Table 1 entries as high as 46.50%. Figure citations in the sensitivity-analysis subsection also appear mismatched against the actual figure captions, and a citation string in the genotype section is garbled, likely from a page-break artifact.

Beyond these, a more substantive point: given I² near 98–99% throughout, a single pooled headline figure (18%) may understate how disparate the underlying data are, even with subgroup analysis performed. Notably, the manuscript’s claim that “one study reported

both viruses” appears incorrect Table 1 shows two (El Qazoui et al., 2024 and Gopalkrishna et al., 2021, each listed under separate norovirus/rotavirus rows). We would also flag that nearly half of included studies (20/45) originate from India, which limits generalizability to other LMIC regions; that the English-only search restriction may have compounded the detected publication bias by excluding French/Spanish/Portuguese-language literature; and that pooling cross-sectional and surveillance designs together, despite differing methodologies, risks obscuring real differences in burden. Finally, if the Data Availability Statement restricts data to “available upon request,” this would not meet PLOS ONE’s minimal-dataset policy we could not verify this wording against the material provided and would ask the editor to confirm with the authors.

None of these issues undermine the manuscript’s core validity or contribution; they are correctable through text and data reconciliation, consistent with Accept with Text Revisions. However, several of the items above are substantive data-accuracy checks, not merely stylistic ones, and should be resolved before production.

2. Line-by-Line Revisions & Language Corrections

Representative examples are given for each manuscript section; this is not an exhaustive list. Citation-bracket spacing (e.g., “word[12]” instead of “word [12]”) recurs dozens of times throughout and should be corrected globally.

Response: All language-related concerns have been corrected in accordance with your comments. Please see the attached “Response to Reviewers” document for detailed responses to each comment.

Attachment Submitted filename: Response to Reviewers.docx

# Peer review: Decision Letter 1

**DOI:** 10.1371/journal.pone.0359886.r003

**Authors:** Ricardo Q. Gurgel

15 Sep 2026

Dear Dr. Ayele,

Thank you for submitting your manuscript to PLOS One. After careful consideration, we feel that it has merit but does not fully meet PLOS One’s publication criteria as it currently stands. Therefore, we invite you to submit a revised version of the manuscript that addresses the points raised during the review process.

plosone@plos.org. When you're ready to submit your revision, log on to https://www.editorialmanager.com/pone/ and select the 'Submissions Needing Revision' folder to locate your manuscript file.

- A letter that responds to each point raised by the academic editor and reviewer(s). You should upload this letter as a separate file labeled 'Response to Reviewers'.
- A marked-up copy of your manuscript that highlights changes made to the original version. You should upload this as a separate file labeled 'Revised Manuscript with Track Changes'.
- An unmarked version of your revised paper without tracked changes. You should upload this as a separate file labeled 'Manuscript'.

If you would like to make changes to your financial disclosure, please include your updated statement in your cover letter. Guidelines for resubmitting your figure files are available below the reviewer comments at the end of this letter.

If applicable, we recommend that you deposit your laboratory protocols in protocols.io to enhance the reproducibility of your results. Protocols.io assigns your protocol its own identifier (DOI) so that it can be cited independently in the future. For instructions see: https://journals.plos.org/plosone/s/submission-guidelines#loc-laboratory-protocols. Additionally, PLOS One offers an option for publishing peer-reviewed Lab Protocol articles, which describe protocols hosted on protocols.io. Read more information on sharing protocols at https://plos.org/protocols?utm_medium=editorial-email&utm_source=authorletters&utm_campaign=protocols.

As the corresponding author, your ORCID iD is verified in the submission system and will appear in the published article. PLOS supports the use of ORCID, and we encourage all coauthors to register for an ORCID iD and use it as well. Please encourage your coauthors to verify their ORCID iD within the submission system before final acceptance, as unverified ORCID iDs will not appear in the published article. *Only*  the individual author can complete the verification step; PLOS staff *cannot*  verify ORCID iDs on behalf of authors.

We look forward to receiving your revised manuscript.

Kind regards,

Ricardo Q. Gurgel, PhD

Academic Editor

PLOS One

Journal Requirements:

If the reviewer comments include a recommendation to cite specific previously published works, please review and evaluate these publications to determine whether they are relevant and should be cited. There is no requirement to cite these works unless the editor has indicated otherwise.

Please review your reference list to ensure that it is complete and correct. If you have cited papers that have been retracted, please include the rationale for doing so in the manuscript text, or remove these references and replace them with relevant current references. Any changes to the reference list should be mentioned in the rebuttal letter that accompanies your revised manuscript. If you need to cite a retracted article, indicate the article’s retracted status in the References list and also include a citation and full reference for the retraction notice.

Additional Editor Comment:

Please do these changes and the manuscript will be accetpted. The changes are: My recommendation: Accept with following minor revision : Most changes are satisfactory; however, please (1) submit a clean file free of tracked-changes artifacts, (2) reconcile the I² value throughout, (3) either add the promised diagnostic-method meta-regression or remove that from the response letter, and (4) proofread the specific lines noted, several of which were marked 'Corrected' but remain unchanged.

[Note: HTML markup is below. Please do not edit.]

Reviewers' comments:

Reviewer's Responses to Questions

**Comments to the Author**

Reviewer #1: All comments have been addressed

Reviewer #2: (No Response)

**********

2. Is the manuscript technically sound, and do the data support the conclusions?

Reviewer #1: Yes

Reviewer #2: Yes

**********

3. Has the statistical analysis been performed appropriately and rigorously?

Reviewer #1: Yes

Reviewer #2: Yes

**********

4. Have the authors made all data underlying the findings in their manuscript fully available?

The PLOS Data policy

Reviewer #1: Yes

Reviewer #2: Yes

**********

5. Is the manuscript presented in an intelligible fashion and written in standard English?

Reviewer #1: Yes

Reviewer #2: (No Response)

**********

Reviewer #1: I thank the authors for their careful consideration of my comments and for the revisions made to the manuscript. After reviewing the authors’ responses and the revised manuscript, I consider that my concerns have been adequately addressed.

The revisions have improved the clarity, methodological rigor, and overall quality of the manuscript. I have no further major comments or concerns.

I therefore recommend acceptance of the manuscript in its revised form.

Reviewer #2: My recommendation: Accept with following minor revision : Most changes are satisfactory; however, please (1) submit a clean file free of tracked-changes artifacts, (2) reconcile the I² value throughout, (3) either add the promised diagnostic-method meta-regression or remove that from the response letter, and (4) proofread the specific lines noted, several of which were marked 'Corrected' but remain unchanged.

**********

what does this mean?). If published, this will include your full peer review and any attached files.

If you choose “no”, your identity will remain anonymous but your review may still be made public.

**Do you want your identity to be public for this peer review?**  For information about this choice, including consent withdrawal, please see our Privacy Policy

Reviewer #1: **Yes:**  CATHARINA CORREA COSTA

Reviewer #2: No

**********

[NOTE: If reviewer comments were submitted as an attachment file, they will be attached to this email and accessible via the submission site. Please log into your account, locate the manuscript record, and check for the action link "View Attachments". If this link does not appear, there are no attachment files.]

To ensure your figures meet our technical requirements, please review our figure guidelines: https://journals.plos.org/plosone/s/figures

You may also use PLOS’s free figure tool, NAAS, to help you prepare publication quality figures: https://journals.plos.org/plosone/s/figures#loc-tools-for-figure-preparation.

NAAS will assess whether your figures meet our technical requirements by comparing each figure against our figure specifications.

# Author comment: Author response to Decision Letter 2

**DOI:** 10.1371/journal.pone.0359886.r004

15 Sep 2026

Response to Reviewers

We sincerely thank the reviewers and the Academic Editor for their careful evaluation of our revised manuscript and for their constructive and valuable comments. We greatly appreciate Reviewer 1’s recommendation for acceptance and Reviewer 2’s positive assessment of the revised manuscript. We have carefully considered the remaining minor issues raised by Reviewer 2 and have addressed each point as detailed below.

Response to Reviewer 2

Comment 1:

“Please submit a clean file free of tracked-changes artifacts.”

Response: Thank you for pointing this out. We have carefully reviewed the revised manuscript and prepared a clean version free of tracked changes, comments, and other revision artifacts. The clean manuscript has been uploaded as requested.

Comment 2:

“Reconcile the I² value throughout.”

Response: Thank you for raising this point. We carefully checked all reported I² values throughout the manuscript. We confirmed that the differences in I² values do not represent inconsistencies or errors; rather, they reflect the different levels of heterogeneity observed across the overall meta-analysis and the various subgroup analyses. Because each subgroup analysis is based on a different set of studies, the corresponding I² values naturally differ. We have nevertheless carefully reviewed the presentation of all I² values throughout the manuscript and ensured that each value corresponds correctly to its respective overall or subgroup analysis and is reported consistently with the statistical results.

Comment 3:

“Either add the promised diagnostic-method meta-regression or remove that from the response letter.”

Response: We sincerely thank the reviewer for this important clarification and apologize for any confusion caused by our previous response. Following the reviewer’s suggestion, we have removed the statement regarding the promised diagnostic-method meta-regression from the response letter. We have also carefully reviewed the manuscript and confirmed that it contains no unsupported claims regarding this analysis. We greatly appreciate the reviewer’s careful attention to this matter, which has helped us improve the clarity and accuracy of our response.

Comment 4:

“Proofread the specific lines noted, several of which were marked ‘Corrected’ but remain unchanged.”

Response: We sincerely appreciate the reviewer for identifying this issue. We have rechecked all the specific lines and comments raised in the previous review round, particularly those for which we had indicated “Corrected.” We carefully compared the reviewer’s comments, our previous responses, and the revised manuscript, and have now made the necessary corrections wherever they were still missing. The manuscript has also been thoroughly proofread to ensure that all stated corrections are accurately reflected in the final version.

Attachment Submitted filename: 2 Response to Reviewers.docx

# Editor report: Decision Letter 2

**DOI:** 10.1371/journal.pone.0359886.r005

**Authors:** Ricardo Q. Gurgel

20 Sep 2026

Prevalence and Genotype Distribution of Norovirus and Rotavirus Among Children Under Five with Diarrheal Infection in Lower-Middle-Income Countries After Rotavirus Vaccine Introduction: A Systematic Review and Meta-analysis

PONE-D-26-34325R2

Dear Dr. Ayele,

We’re pleased to inform you that your manuscript has been judged scientifically suitable for publication and will be formally accepted for publication once it meets all outstanding technical requirements.

Within one week, you’ll receive an e-mail detailing the required amendments. When these have been addressed, you’ll receive a formal acceptance letter and your manuscript will be scheduled for publication.

An invoice will be generated when your article is formally accepted. Please note, if your institution has a publishing partnership with PLOS and your article meets the relevant criteria, all or part of your publication costs will be covered. Please make sure your user information is up-to-date by logging into Editorial Manager at Editorial Manager® and clicking the ‘Update My Information' link at the top of the page. For questions related to billing, please contact billing support.

If your institution or institutions have a press office, please notify them about your upcoming paper to help maximize its impact. If they’ll be preparing press materials, please inform our press team as soon as possible -- no later than 48 hours after receiving the formal acceptance. Your manuscript will remain under strict press embargo until 2 pm Eastern Time on the date of publication. For more information, please contact onepress@plos.org.

Kind regards,

Ricardo Q. Gurgel, PhD

Academic Editor

PLOS One

Additional Editor Comments (optional):

Reviewers' comments:

# Editor report: Acceptance letter

**DOI:** 10.1371/journal.pone.0359886.r006

**Authors:** Ricardo Q. Gurgel

PONE-D-26-34325R2

PLOS One

Dear Dr. Ayele,

I'm pleased to inform you that your manuscript has been deemed suitable for publication in PLOS One. Congratulations! Your manuscript is now being handed over to our production team.

At this stage, our production department will prepare your paper for publication. This includes ensuring the following:

* All references, tables, and figures are properly cited

* All relevant supporting information is included in the manuscript submission,

* There are no issues that prevent the paper from being properly typeset

You will receive further instructions from the production team, including instructions on how to review your proof when it is ready. Please keep in mind that we are working through a large volume of accepted articles, so please give us a few days to review your paper and let you know the next and final steps.

Lastly, if your institution or institutions have a press office, please let them know about your upcoming paper now to help maximize its impact. If they'll be preparing press materials, please inform our press team within the next 48 hours. Your manuscript will remain under strict press embargo until 2 pm Eastern Time on the date of publication. For more information, please contact onepress@plos.org.

You will receive an invoice from PLOS for your publication fee after your manuscript has reached the completed accept phase. If you receive an email requesting payment before acceptance or for any other service, this may be a phishing scheme. Learn how to identify phishing emails and protect your accounts at https://explore.plos.org/phishing.

If we can help with anything else, please email us at customercare@plos.org.

Thank you for submitting your work to PLOS One and supporting open access.

Kind regards,

PLOS One Editorial Office Staff

on behalf of

Professor Ricardo Q. Gurgel

Academic Editor

PLOS One
